# New gold standard: weakly capped infant Au nanoclusters with record high catalytic activity for 4-nitrophenol reduction and hydrogen generation from an ammonia borane–sodium borohydride mixture†

**DOI:** 10.1039/d0na00639d

**Published:** 2020-09-28

**Authors:** Dinabandhu Patra, Srinivasa Rao Nalluri, Hui Ru Tan, Mohammad S. M. Saifullah, Ramakrishnan Ganesan, Balaji Gopalan

**Affiliations:** Department of Chemistry, Birla Institute of Technology and Science (BITS) Pilani Hyderabad Campus, Jawahar Nagar, Kapra Mandal Hyderabad-500078 India ram.ganesan@hyderabad.bits-pilani.ac.in gbalaji@hyderabad.bits-pilani.ac.in; Institute of Materials Research and Engineering, A*STAR (Agency for Science, Technology, and Research) 2 Fusionopolis Way, #08-03 Innovis Singapore 138634 Singapore saifullahm@imre.a-star.edu.sg

## Abstract

Increasing the surface area-to-volume ratio of materials through size reduction is a desired approach to access maximum possible surface sites in applications such as catalysis. However, increase in the surface energy with the decrease in dimension warrants strong ligands to stabilize nanosystems, which mask the accessibility of the active surface sites. Owing to this, the realization of the true potential of a catalyst's surface remains challenging. Here, we employed a rationally designed strategy to synthesize infant Au nanoclusters—that alleviates the requirement of any separate ligand removal step—to unleash their actual potential to register a record high maximum turn-over frequency (TOF_max_) of 72 900 h^−1^ and 65 500 h^−1^ in the benchmark catalytic reduction of 4-nitrophenol and catalytic H_2_ generation from an ammonia borane–sodium borohydride mixture, respectively. Such a phenomenal catalytic activity has been realized *via* the synthesis and stabilization of Au nanoclusters using solid citric acid and a super-concentrated aqueous AuCl_3_ solution, a pathway entirely different from the conventional modifications of the Turkevich and Brust methods. The crux of the synthetic strategy lies in precise control of the water content and thereby ensuring that the final Au nanoclusters remain in the solid state. During the synthesis, citric acid not only acts as a reducing agent to yield ‘infant’ Au nanoclusters but also provides a barrier matrix to arrest their growth. In solution, its weak capping ability and rapid dissolution allows the reactants to easily access the active sites of Au nanoclusters, thus leading to faster catalysis. Our study reveals that the true potential of metal nanoclusters as catalysts is actually far higher than what has been reported in the literature.

## Abstract 2

Active sites are preserved in the citric acid-capped Au nanoclusters prepared in solid state. In water, the rapid dissolution of citric acid allows the reactants to easily access the active sites of infant Au nanoclusters leading to faster catalysis.

## Graphical abstract

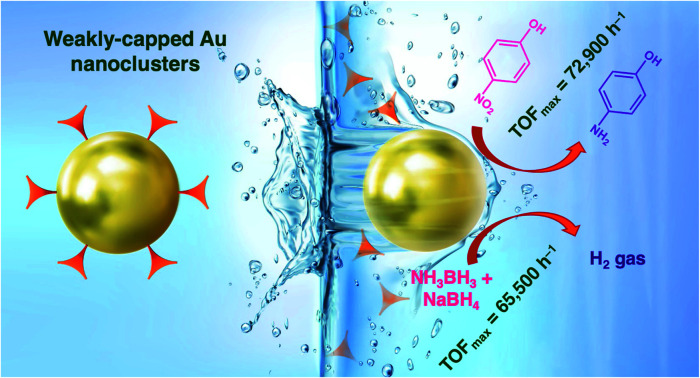

## Introduction

The quest for realizing the true potential of metal catalysts has been an on-going effort. Several classes of materials such as supported porous catalysts, non-supported nanosystems, and single atom supported catalysts have been rigorously employed to maximize the catalytic efficacy.^1–3^ Non-supported nanosystems including metal nanoparticles and nanoclusters have attracted huge attention due to their high surface area-to-volume ratio.^4–6^ However, creating particles that are sufficiently small is challenging due to the effects arising from high surface energy. Such a high surface energy warrants strong ligands to stabilize the nanosystems, which typically creates a firm binding of the ligand at the surface of the catalyst and thereby masks the active surface sites.

Gold has been regarded as an invaluable catalyst.^7–9^ The synthesis of Au-based nanosystems has become an intensive area of research for various catalytic conversions.^10,11^ Plasmonic Au nanoparticles are most commonly synthesized through the Brust and Turkevich methods.^12,13^ On the other hand, the non-plasmonic Au nanoclusters (sub-2 nm) are prepared through modified Brust (bottom-up) or ligand mediated etching (top-down) methods.^6,14–17^ In both these approaches, the Au precursor and reducing agent are used in the solution phase. The natural inclination to synthesize Au nanoclusters is to use (i) highly dilute (in mM concentrations) Au precursor solution, (ii) strong surface stabilizing agents (thiol- and/or phosphine-based ligands), and (iii) low temperatures to avoid coalescence of the formed Au nanoclusters.^18–22^ Fundamentally, the chemical synthesis of metal nanoparticles/nanoclusters involves nucleation and growth. Arresting the growth in solution media, specifically to obtain non-ligated metal nanoclusters, is challenging due to the lack of barrier for diffusion of the nucleates. Reports are available that describe the synthesis of atom-precise strongly ligated Ag nanoclusters through the solid state approach.^23,24^ Inexorably, the availability of surface active sites of the nanoclusters obtained through the existing synthetic approaches is highly deprived and thereby applications including catalysis and sensing that rely on the accessible surface active sites suffer from realizing the actual efficacy. Furthermore, due to the steric factor of the ligands, the Au atoms adjacent to the ligand-coordinated centers also become inaccessible, particularly in catalysis. This realization has prompted researchers to look for strategies to remove or vary the surface ligands to enhance the catalytic activity.^6,25–28^ Hutchings *et al.* have shown that gold nanoparticles stabilized with polyvinyl alcohol supported on TiO_2_ upon subjecting to ligand removal through refluxing in an organic solvent enhances the catalytic activity, while retaining the size and morphology of the catalysts.^29^ Based on the above discussion, the problem can be stated as: “how to prepare stable Au nanoclusters without compromising the surface active sites?” The solution to this problem warrants an unconventional synthetic approach. We have addressed this challenge through a designed and sustainable synthetic strategy, which is not a simple modification of any of the methods reported in the literature. Here, we have taken a very high and precise concentration of Au precursor aqueous solution (12% of solvent, 24.9 M) of gold(iii) chloride (AuCl_3_) along with solid citric acid as the reducing agent. The resultant mixture was ground well in a mortar and pestle to obtain ‘infant Au nanoclusters’ of the size ∼1–2.5 nm. We found that these nanoclusters exhibited a record high catalytic activity with a maximum turn-over frequency (TOF_max_) of 72 900 h^−1^, as opposed to the state-of-the-art record high TOF of 9000 h^−1^ for Au-based catalysts towards the benchmark reduction of 4-nitrophenol.^30^ In the case of hydrogen generation from an ammonia borane–sodium borohydride mixture, a value of 65 500 h^−1^ was observed, which is also the highest for any Au based systems.

## Experimental section

### Materials and methods

Gold(iii) chloride was purchased from Alfa-Aesar, while 2-mercaptobenzimidazole (2-MBI) was procured from Sigma-Aldrich. Anhydrous citric acid, anhydrous tartaric acid, anhydrous ascorbic acid, sodium thiosulfate, 4-nitrophenol, and sodium borohydride (NaBH_4_) were purchased from SD Fine Chemicals (India). All the reagents were used without any further purification. Ammonia borane was synthesized by following a literature procedure.^31^ Millipore water was used in every solution preparation, titration, and catalytic reaction.

#### Preparation of super concentrated AuCl_3_ solution

The AuCl_3_ solution used in the synthesis of Au nanoclusters was prepared by exposing 25 mg of AuCl_3_ to humidity in a controlled chamber (*R*_H_ value of ∼80%) for 24 h. This exposure resulted in the natural hydration of AuCl_3_ to yield a homogeneous solution. The mass of the solution was periodically monitored until no change in mass was observed. We found that a constant mass was attained after 12–16 h of exposure to humidity. The amount of AuCl_3_ present in the solution was estimated through the titration against a standardized hypo solution, which revealed ∼88% of AuCl_3_ by weight. Such a solution (density = 2.4 g mL^−1^) containing ∼12% of water is phenomenally concentrated and hence we termed it super-concentrated AuCl_3_ solution.

#### Synthesis of Au nanoclusters

150 mg of anhydrous citric acid was taken in a clean mortar, to which a calculated amount of highly concentrated aqueous AuCl_3_ solution (containing 12 wt% of water) was added. The weight of AuCl_3_ solution was varied as 1.5, 3, 6, 18, and 36 mg against a fixed quantity of 150 mg of anhydrous citric acid powder and the resultant mixtures were denoted as ACA-0.25, ACA-0.5, ACA-1, ACA-3, and ACA-6, respectively. Each composition was ground with a pestle for a duration of 10 min, unless otherwise mentioned. The critical aspect of the synthetic procedure is the optimal amount of water required for the reduction, which is available from the highly concentrated AuCl_3_ precursor solution. The relative humidity (*R*_H_) of the laboratory was maintained below 55%, above which the conditions are conducive for the growth of Au nanoclusters into Au nanoparticles. The as-prepared Au nanoclusters were used without any further purification for characterization and catalytic reduction of 4-nitrophenol and hydrogen generation from the ammonia borane–NaBH_4_ mixture.

### Characterization

Scanning transmission electron microscopy-high angle annular dark field imaging (STEM-HAADF, FEI Titan, 200 kV) was performed to characterize the size and morphology of the as-synthesized Au nanoclusters. Drop-casting of the as-synthesized Au nanoclusters in aqueous solution on the copper grids affected the size and morphology due to the growth dynamics in solution and during the subsequent drying process (Fig. S20†). This challenge was overcome by directly placing the solid citric acid stabilized Au nanoclusters over the transmission electron microscopy grids containing holey carbon films with gentle dabbing followed by shaking off to get rid of the loose samples. Under 200 kV electron beam irradiation, the larger grains of citric acid on the holey carbon grid were observed to melt. However, the thinner regions of citric acid were found to retain the sample integrity and hence those were used for imaging. X-ray photoelectron spectroscopy (XPS, K-Alpha, Thermo-Fischer) equipped with monochromated Al-K_α_ radiation was used to study the changes in the oxidation state of gold. The combination of a low power X-ray source (72 W) and the narrow scan measurements prior to the survey scan has been employed in order to avoid any radiation induced *in situ* reduction of AuCl_3_. The obtained XPS data were analysed using Avantage software. The data were deconvoluted for Au^3+^ and Au^0^ species by fixing the spin–orbit coupling value as 3.7 eV. The reduction of AuCl_3_ was additionally monitored through redox titration using standardized sodium thiosulfate solution. First, a stock solution containing 1 mg of AuCl_3_ in 50 mL of water was prepared, from which 2 mL of solution was mixed with a mixture containing 20 mg KI and 5 mL starch solution. The resulting mixture was then titrated against 0.625 mM hypo solution. In a similar manner, by taking appropriate quantities, the titration of ACA samples was also performed. Solid state diffuse reflectance spectroscopy (DRS, JASCO V-670) measurements in the 200–1000 nm wavelength range at a scan speed of 400 nm min^−1^ were recorded to follow the UV-visible spectral profiles of the Au nanoclusters. For all the DRS measurements, a barium sulfate window was used as the reference. Powder X-ray diffraction (XRD, Rigaku Ultima-IV) measurements were performed for the as-synthesized samples in the range of 30°–80° at a scan speed 0.5° min^−1^ to follow the reduction of the AuCl_3_ precursor yielding crystalline metallic gold. Time-dependent XRD studies were performed in the narrow 2*θ* range of 35.5°–39.5° at a scan speed 0.1° min^−1^ to monitor the changes in the intensity of the Au(111) crystal plane. Fourier transform infrared spectroscopy (FT-IR, JASCO FT-IR 4200) was employed to follow the vibrational frequencies with the change in the ratio of citric acid to AuCl_3_ precursor. The reduction of 4-nitrophenol was followed by time-dependent UV-visible spectroscopy using an Ocean Optics DH-Mini spectrometer. The UV-visible spectral data of the 4-nitrophenol reduction were recorded at a rate of 9 scans per second and the data after every ninth scan were used for analysis after the necessary background correction. For the aggregation dynamics studies using UV-visible spectroscopy, with 1000 mg L^−1^ of ACA-1 catalyst solution no visible color change was observed up to 120 min. Hence, the initial concentration of 3000 mg L^−1^ of ACA-1 catalyst solution was used. With respect to the Au content in this solution, the initial concentration of other ACA catalyst solutions was fixed. Liquid chromatography (LC) coupled with mass spectrometry (Shimadzu LCMS 8040) was employed to monitor the concentration of 2-MBI for surface Au content estimation studies.

### Estimation of available Au surface sites

To determine the available surface active sites in Au nanoclusters, the 2-MBI adsorption method was followed. In a typical experiment, 100 μL of 1000 mg L^−1^ ACA catalyst aqueous solution was treated for 1 h with 2 mL of a 1 : 1 water–methanol mixture containing 40 nmol (6 μg) of 2-MBI. After this, the solution was centrifuged at 13 000 rpm for 30 min. The supernatant solution was then analysed using LC. The decrease in the concentration of 2-MBI with respect to the untreated control is attributed to that adsorbed over the Au surface. For calibration, 100 μL of 1000 mg L^−1^ citric acid solution was added to a 1 : 1 water–methanol mixture containing different concentrations of 2-MBI.

### Catalytic reduction of 4-nitrophenol and cyclability studies

The as-synthesized Au nanoclusters were studied for their catalytic efficacy towards the benchmark 4-nitrophenol reduction reaction. In a typical experiment, a mixture of a freshly prepared solution containing 0.14 mM 4-nitrophenol and 14 mM sodium borohydride was taken in a quartz cuvette equipped with a magnetic stirrer. About 60 μL of 1000 mg L^−1^ ACA-1 catalyst aqueous solution was added into the cuvette containing the reactant mixture. The progress of the reaction was monitored through the decrease in the absorbance at 400 nm wavelength. For cyclability studies, after each cycle, a calculated amount of a solution containing 4-nitrophenol and sodium borohydride was added such that their final overall concentrations are 0.14 and 14 mM, respectively (Table S1†). It can be noted that the total reaction volume from the second cycle onwards increased progressively, and, thus, a gentle increase in the absolute quantity of 4-nitrophenol was seen in subsequent cycles. Another point to note is that excess sodium borohydride along with its by-products and 4-aminophenol formed during the earlier cycles also remain in the reaction medium, which may potentially affect the reaction kinetics from the beginning of the second cycle onwards.

### Catalytic hydrogen production from the ammonia borane–NaBH_4_ mixture

The catalytic hydrogen production from ammonia borane was studied using an ammonia borane and sodium borohydride mixture at a molar ratio 2 : 1, respectively. The experiment was performed by dissolving 10 mg of ammonia borane and 6.2 mg of sodium borohydride in 2 mL water and the resultant solution was added into a round bottom flask containing 50 mg of ACA-1 and kept stirred at 735 rpm. The liberated hydrogen was measured using a gas burette. The order of the reaction with respect to the ammonia borane–sodium borohydride mixture concentration was measured by varying the amount of the hydrogen source (maintaining the 2 : 1 molar ratio of ammonia borane to sodium borohydride) using a constant 50 mg of ACA-1 catalyst. The order with respect to catalyst concentration was determined by maintaining a constant amount of the ammonia borane and sodium borohydride mixture (10 mg of ammonia borane and 6.2 mg of sodium borohydride) dissolved in 2 mL water, and varying the amount of the ACA-1 catalyst. In both these order determination experiments, the reaction contents were stirred at 735 rpm and the rate was calculated from the second point.

## Results and discussion

### Synthesis and characterization of Au nanoclusters

**Fig. 1:**
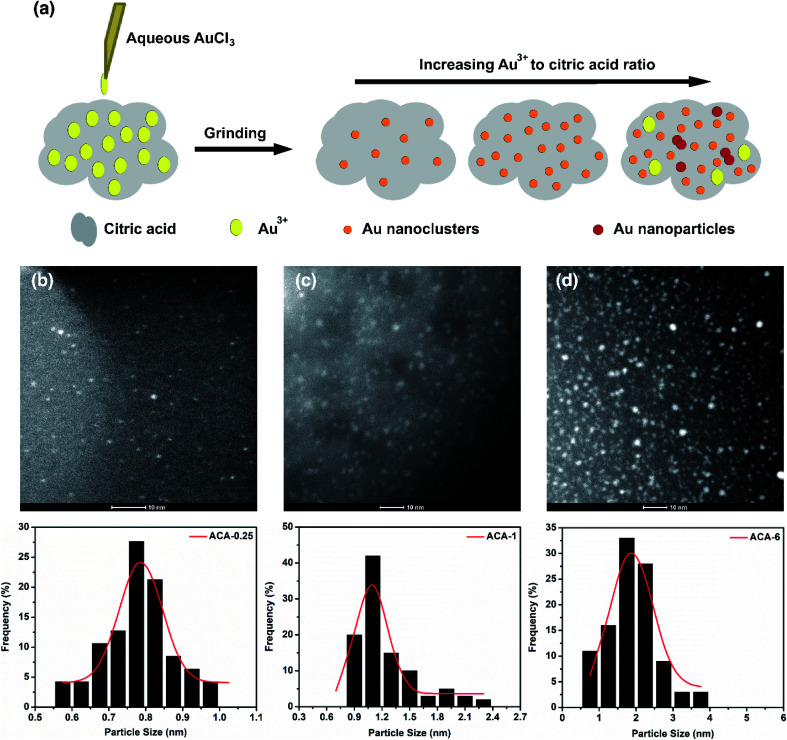
(a) Schematic representation of the solid state grinding process for synthesizing infant Au nanoclusters. STEM-HAADF images of (b) ACA-0.25 (c) ACA-1, and (d) ACA-6. Their corresponding size distribution histograms are given below. The scale bar in STEM-HAADF images corresponds to 10 nm. An increase in the number density of Au nanoclusters with increasing AuCl_3_ to citric acid ratio can be observed.

Fig. 1a shows the schematic representation of the solid state grinding process employed in this work. Five different compositions containing AuCl_3_ and citric acid, namely ACA-0.25, ACA-0.5, ACA-1, ACA-3, and ACA-6 have been synthesized. The numbers 0.25, 0.5, 1, 3, and 6 represent the varying amounts of AuCl_3_ solution in mg, while the quantity of citric acid was fixed as 25 mg in all the cases. It should be noted that each batch of synthesis was scaled up to 150 mg with respect to citric acid. After mixing the appropriate amounts of reactants, the mixture was ground well for 10 min, unless otherwise specified. A gentle change in color from yellow to green was observed, whose intensity was found to increase with increasing gold content. Successful formation of Au nanoclusters was confirmed by high angle annular dark field imaging (HAADF) using an FEI Titan electron microscope operating at 200 kV in the scanning transmission electron microscopy (STEM) mode, as shown in Fig. 1b–d. The respective size distribution plots are shown below the STEM-HAADF images of ACA-0.25, ACA-1, and ACA-6. In general, both the average size and the number density of Au nanoclusters embedded in the citric acid matrix increased with increasing gold content (Table 1). The average size of Au nanoclusters in ACA-0.25, ACA-1.0, and ACA-6 has been found to be 0.8 ± 0.1 nm, 1.0 ± 0.1 nm, and 1.9 ± 0.6 nm, respectively. To ascertain the effect of grinding time, the ACA-1 composition was ground for a total period of 20 min [ACA-1 (20 min)], whose STEM-HAADF imaging revealed a slightly larger cluster size of 1.3 ± 0.1 nm along with an increase in number density, when compared with the 10 min ground sample (Fig. S1a and b†). The uniqueness of this synthetic approach lies in the critical amount of water in the AuCl_3_ solution.

**Table 1:** Average size and standard deviation in size for various synthesized nanoclusters

Sample	Avg. size (nm)	Std dev. (nm)	Polydispersity (%)
ACA-0.25	0.8	0.10	6.5
ACA-1	1.0	0.10	12.8
ACA-6	1.9	0.60	30.8
ACA-1 (20 min)	1.3	0.10	10.3

**Fig. 2:**
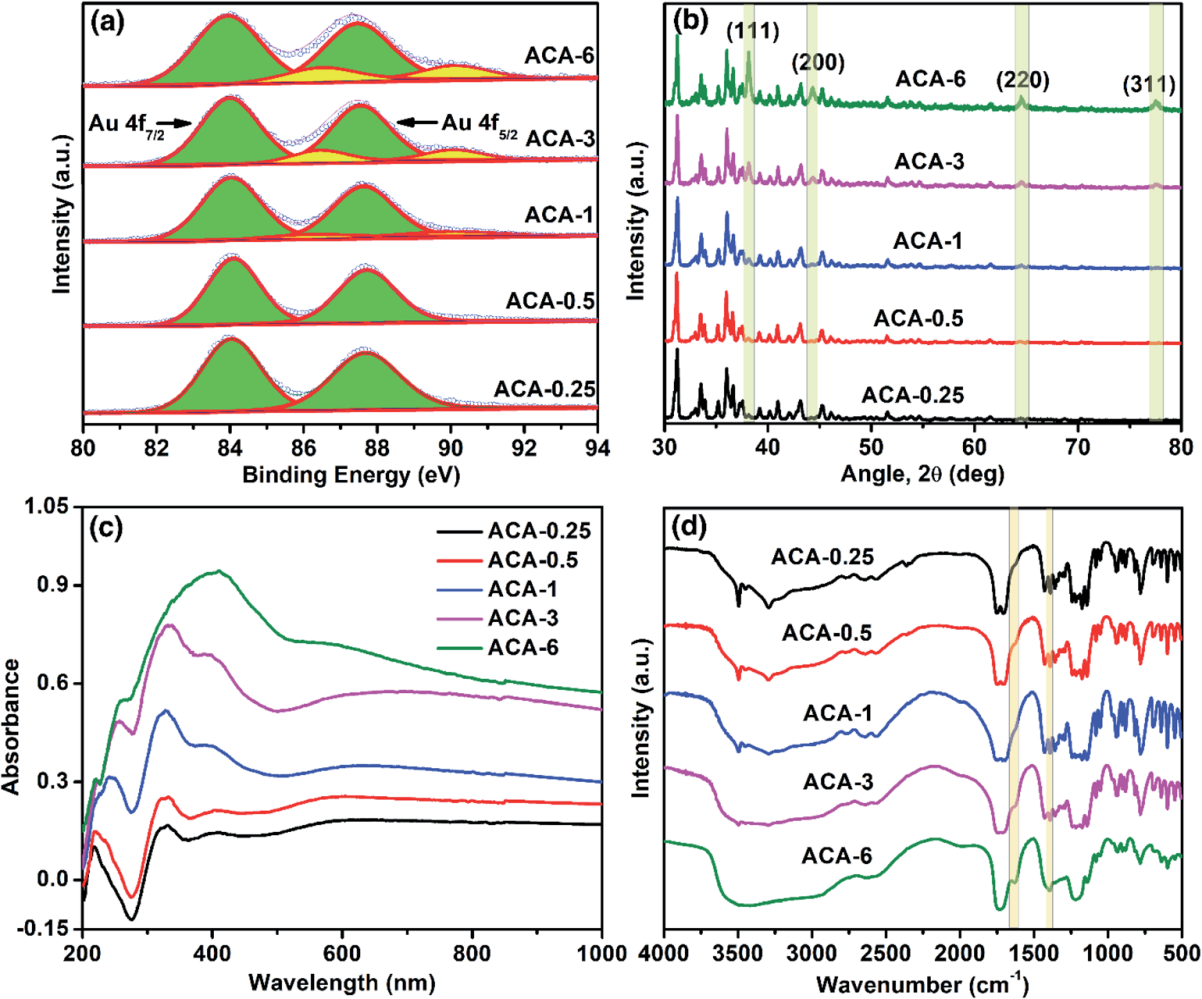
Characterization of ACA samples: (a) narrow scan XPS, (b) XRD, (c) solid state DRS and (d) FT-IR data obtained after 10 min of grinding. In XRD, the peaks corresponding to gold are shaded for clarity, while the other peaks correspond to crystalline citric acid. On the other hand, in FT-IR, the shaded bands correspond to 1628 and 1398 cm^−1^ for *ν*_asym_(COO^−^) and *ν*_sym_(COO^−^) stretching modes, respectively.

X-ray photoelectron spectroscopy (XPS) was used to follow the oxidation state of Au in the synthesized compositions. The progress of the Au^3+^ reduction was monitored both as a function of the initial AuCl_3_ amount and grinding time. The narrow scan XPS measurements of ACA-0.25 to ACA-6 were recorded and the results are presented in Fig. 2a. The binding energy for Au^0^ was observed at 84.0 and 87.7 eV corresponding to Au f_7/2_ and Au f_5/2_ levels, while that for Au^3+^ was observed at 86.5 and 90.2 eV, respectively.^32^ The percentage of Au^0^ present in the case of ACA-0.25, and ACA-0.5 was found to be ∼99%, while that in ACA-1, ACA-3, and ACA-6 was determined to be ∼95, ∼88 and ∼82%, respectively. Thus, with increasing AuCl_3_ content more than that in ACA-1, the percentage of unreduced AuCl_3_ was found to be higher. To follow the reduction kinetics, the XPS spectra of ACA-1 samples at every 2 min interval of grinding were recorded (Fig. S2a†). It was observed that a significant amount to the tune of ∼83% AuCl_3_ got reduced in the first 2 min of grinding. By further increasing the grinding duration up to 10 min, the relative peak intensity of Au^3+^ was found to gradually decrease while that of Au^0^ increased, thereby confirming the progress of reduction with grinding time. Thus, for ACA-1, the required grinding time for the near-complete reduction of AuCl_3_ is 10 min. The percentage reduction of the AuCl_3_ precursor with the grinding time was also verified for samples ACA-1 to ACA-6 by titrating against a standard sodium thiosulfate solution, wherein a good agreement with the XPS results was obtained (Fig. S3, Tables S2 and S3†).^33^ In the case of ACA-1 (20 min), a complete reduction of Au^3+^ to Au^0^ was observed. This indicated that the excessive grinding time with citric acid was effective in the complete reduction of the AuCl_3_ precursor (Fig. S1b†).

X-ray diffraction (XRD) of the as-synthesized ACA samples revealed the presence of face-centered cubic gold (Fig. 2b), indicating the presence of metallic Au^0^. With the increase in the initial gold content, the relative intensity of citric acid was found to decrease, while that of the Au(111) peak at 2*θ* = 38.1° was observed to increase. In addition, the increase in the intensity of the Au(111) peak with increasing gold content complements the sequential increase in the number density of Au nanoclusters observed in STEM-HAADF imaging. Likewise, XRD measurements recorded at different grinding times for ACA-1 revealed a clear increase in the Au(111) peak intensity with respect to time (Fig. S2b†), thus corroborating the progress in AuCl_3_ reduction as observed from XPS. For ACA-1 (20 min), the peak intensity of the Au(111) plane was found to be higher than that observed for ACA-1 (Fig. S1c†). This slight increase in the intensity of Au(111) in ACA-1 (20 min) may be due to the higher crystallinity as a result of the extended grinding process.

Optical property analysis is powerful in differentiating the plasmonic nanoparticles and non-plasmonic nanoclusters of gold. Solid state diffuse reflectance UV-visible spectroscopy (DRS) measurements were performed to understand the optical behavior of the synthesized samples. The solid state DRS spectra (Fig. 2c) of the ACA samples revealed the presence of three distinct features at around 330, 390, and a shallow peak in the range of 590–650 nm; those features were absent in citric acid and AuCl_3_ (Fig. S1e†). The peak values in DRS for the ACA samples agree closely with those reported for strongly ligated Au nanoclusters in the literature.^15^ In particular, the peak at around 300–330 nm is reported to be a characteristic feature of Au nanoclusters.^34^ With increasing gold content, the absorbance was found to increase which could be attributed to the higher absorbance by the progressively increasing number density of Au nanoclusters. In the case of ACA-6, the characteristic peak at 330 nm overlapped with the broad absorbance signal at 400 nm from a significant amount of unreduced AuCl_3_. In the DRS spectra recorded at different grinding times for ACA-1, the absorbance at 330 nm showed a little change, but a significant increase in the wavelength range of 500 to 1000 nm was observed (Fig. S2c†). In addition, the peak at 590–650 nm exhibited a gentle blue shift with increasing the grinding time up to 10 min. All the spectral features observed for ACA-1 samples were also present in ACA-1 (20 min), except a gentle blue shift of the shallow peak to less than 580 nm (Fig. S1d†). The observations from XPS, XRD, and DRS analyses suggest that, in addition to the progress of AuCl_3_ reduction, the growth of already formed Au nanoclusters also takes place simultaneously during the grinding process. It can be surmised from the DRS studies that the position of the surface plasmon resonance peak is resultant of a complex combination of nanocluster size and crystallinity, which in turn may depend on the amount of citric acid and grinding time.

Fourier Transform Infrared Spectroscopy (FT-IR) measurements were performed with respect to the initial gold precursor content (Fig. 2d). It should be noted that the molar ratio of citric acid to Au in ACA-6 is 7.5, while that in ACA-0.25 is 180. The FT-IR spectrum of ACA-0.25 was almost similar to that of pure citric acid, as characterized by the two carbonyl stretching bands at 1751 and 1707 cm^−1^. With a sequential decrease in the citric acid content, a systematic evolution of two bands at 1628 and 1398 cm^−1^ was observed corresponding to the metal-chelated *ν*_asym_(COO^−^) and *ν*_sym_(COO^−^) stretching modes, respectively. This suggests the surface chelation of the carboxylate functionality to the Au nanoclusters. The separation of 230 cm^−1^ between the asymmetric and symmetric stretching modes indicates the η^1^-type of COO^−^ binding.^35^ The source of COO^−^ could be citric acid or any of its oxidized products such as 1,3-acetonedicarboxylate, acetoacetate, *etc.*^36^

The formation of Au nanoclusters can be understood by the conventional nucleation and growth mechanism.^37^ However, our synthetic approach departs from the conventional protocol with the emphasis on the little and precise amount of water needed for the salt solution. This small amount of water is essential to facilitate the distribution of Au^3+^ ions in the solid matrix evenly and also to generate citrate ions from citric acid through dissociation, which eventually reduces Au^3+^ to Au^0^.^38^ More importantly, the smaller the amount of water retained in the solid citric acid matrix, the higher the energy barrier for any diffusion and possible growth of the formed Au nanoclusters.^39^ Thus, solid citric acid acts both as a reducing agent to synthesize Au nanoclusters as well as a barrier matrix to arrest their further growth. In this manner, controlled growth leads to the formation of Au nanoclusters in high yield.

### Catalytic activity and stability of Au nanoclusters towards 4-nitrophenol reduction

**Fig. 3:**
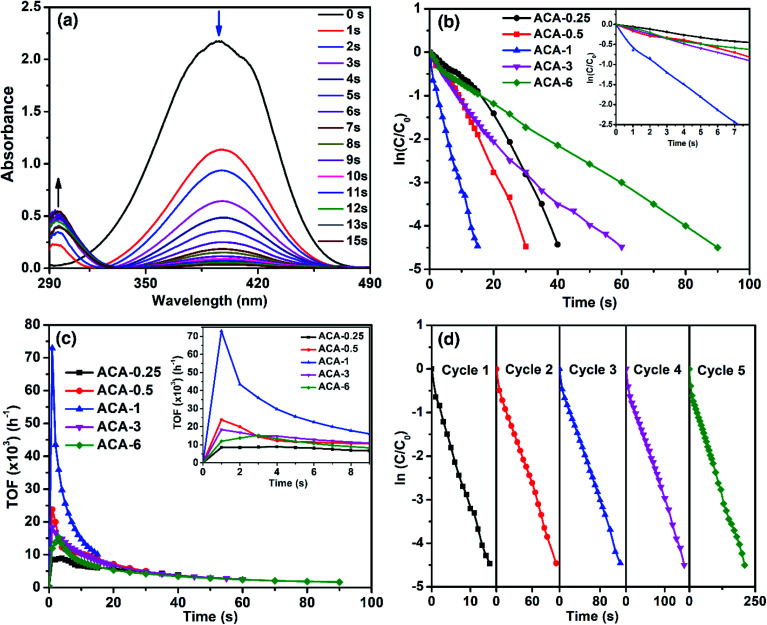
(a) Time-resolved UV-visible spectral profiles of 4-nitrophenol reduction with sodium borohydride in the presence of the ACA-1 catalyst. (b) Plot of ln(*C*/*C*_0_) *versus* time for all ACA samples for the benchmark 4-nitrophenol reduction reaction (the amount of gold was maintained the same in all reactions). (c) Plot of TOF as a function of reaction time for the corresponding data in (b). (d) Cyclability studies up to 5 cycles of 4-nitrophenol reduction using the ACA-1 catalyst.

As the significance of a catalyst lies in its efficiency through the maximum accessible surface active sites, we now demonstrate how the nanoclusters synthesized using this precisely controlled procedure lead to very high catalytic activity. For this, the as-synthesized Au nanoclusters were studied for the catalytic reduction of 4-nitrophenol to 4-aminophenol by NaBH_4_, which is a well-studied system and considered to be one of the benchmark reactions in the literature.^40–42^ The reaction was characterized by the decrease in the absorbance at 400 nm with a concomitant appearance of a peak at 300 nm due to the conversion of 4-nitrophenol to 4-aminophenol (Fig. 3a). For all the catalysis experiments, the amount of Au for each reaction was maintained at 6.7 nmol. That is, with ACA-1, 60 μL of the catalyst solution was used for each reaction from a stock solution of 1000 mg L^−1^. The reaction was screened with different initial concentrations of 4-nitrophenol and NaBH_4_ using the ACA-1 catalyst to obtain suitable conditions for the study. Fig. S4† shows the apparent rate constant dependence on the concentration of the reactants. The data can be explained using the Langmuir–Hinshelwood mechanism, wherein both the reactants adsorb and react at the surface of the particles. For both reactants, the adsorption is reversible and obeyed the Langmuir isotherm. While the diffusion and adsorption/desorption processes for reactants and products are fast, the reduction of adsorbed 4-nitrophenol by surface hydrogen species is the rate determining step. Based on these, for all the reactions, the concentrations of 4-nitrophenol and NaBH_4_ with respect to the initial volume have been fixed as 0.14 and 14.0 mM, respectively. The kinetics of the reaction were analyzed using the pseudo first order model with respect to 4-nitrophenol. Fig. 3b shows the plot of ln(*C*/*C*_0_) *versus* time, where *C*_0_ and *C* represent the 4-nitrophenol concentration at the start of the reaction and at time *t*, respectively. A control experiment with AuCl_3_ was also performed by adding an appropriate quantity of AuCl_3_ solution to the mixture of 4-nitrophenol and NaBH_4_ (Fig. S5†). The *in situ* produced Au nanoparticles in the control experiment took 120 s for the completion of the reaction. In addition, under our experimental conditions, the control experiment exhibited an induction period of 3 s before the onset of the catalytic reduction, which is similar to the observations in the literature reports.^43^ Interestingly, no induction period was observed with any ACA catalysts. In the case of the ACA samples, ACA-6 required 90 s for the completion of the reaction, while ACA-1 required only 15 s for the same (see the video for ACA-1). Thus, a clear trend of enhancement in the catalytic activity was observed with a decrease in the initial gold content up to ACA-1. With a further decrease in the initial gold content, as in ACA-0.5 and ACA-0.25, the time for completion of reaction was found to slightly increase to 30 s and 40 s, respectively. The trend in the catalytic activity can be ascertained by considering the following two factors: percentage of unreduced AuCl_3_, and the citric acid content. In the case of ACA-6, the unreduced AuCl_3_ was as high as 18%, while in ACA-1 it was <5%. Even though the unreduced AuCl_3_ would get reduced *in situ*, the catalytic activity of such particles was found to be less, as observed from the control experiment. Furthermore, the amount of citric acid is less in ACA-6, due to which the coalescence of Au nanoclusters in the reaction solution could be faster than that in ACA-1. Besides, the excess amount of citric acid in the case of ACA-0.25 was found to adversely affect the reaction rate, despite having a very close particle size to ACA-1. Thus, the cumulative factors of optimal citric acid content and higher conversion of the AuCl_3_ to Au nanoclusters could be attributed to the extraordinarily high catalytic efficiency of ACA-1. In the case of ACA-0.5 and ACA-0.25, although the percentage reduction of AuCl_3_ is comparable to ACA-1, the decrease in the catalytic activity can be attributed to the progressively increasing citric acid content that would most likely sterically hinder 4-nitrophenol and NaBH_4_ from approaching and accessing the surface active sites of the Au nanoclusters.

Turn-over frequency (TOF) is a valuable parameter to ascertain the catalytic efficacy. Table 2 summarizes the important catalytic parameters such as TOF_max_, average TOF (TOF_avg_) for the first 10 s, and overall apparent rate constant (*k*_app_). For clarity, the overall rate constant is also expressed in terms of catalyst amount, namely, ‘mass activity parameter’ (*k*_c,g_) and ‘concentration activity parameter’ (*k*_c,m_). Fig. 3c shows the TOF as a function of reaction time. The TOF_max_ determined for ACA-1 was 72 900 h^−1^, which is a record-high value reported for any Au-based catalyst systems.^30^ The TOF_avg_ and the minimum TOF (calculated at near-completion) for ACA-1 were determined to be 14 690 and 10 080 h^−1^, respectively. It must be emphasized that this minimum TOF value is still higher than that reported for any Au-based nanosystems.^30,43^ Such a high catalytic activity can be attributed to the highly surface active Au nanoclusters. It can be seen from Fig. 3c that the TOF reaches a maximum value for all the catalysts in 1 s and then starts to fall off with increasing time. The TOF_max_ for the catalysts followed a trend ACA-0.25 < ACA-6 < ACA-3 < ACA-0.5 < ACA-1. Although the Au content is normalized in all the reactions, the amount of citric acid is different in each composition, which could have influenced the coalescence dynamics of Au nanoclusters and reactants' diffusion to the active sites in the early stage itself. It is to be noted that the unreduced AuCl_3_ precursor in ACA-3 and ACA-6 could also have affected the coalescence dynamics of Au nanoclusters during the early stage of NaBH_4_-mediated *in situ* reduction.

**Table 2:** Summary of the catalyst compositions and their catalytic parameters towards 4-nitrophenol reduction^b^

Sample	Mole ratio of Au/citric acid	Percentage reduction after 10 min of grinding	TOF_max_ (h^−1^)	TOF_avg_^a^ (h^−1^)	*k*_app_ (s^−1^)	*k*_c,g_ (L g^−1^ s^−1^)	*k*_c,m_ (L mol^−1^ s^−1^)
ACA-0.25	1 : 180	>99	8905	6690	0.1409	207.2	40 781
ACA-0.5	1 : 90	>99	23 820	10 290	0.1572	232.9	45 897
ACA-1	1 : 45	95	72 900	14 690	0.285	445.3	88 153
ACA-3	1 : 15	88	18 400	10 340	0.1009	165.4	32 664
ACA-6	1 : 7.5	82	15 245	7890	0.0611	111.1	21 821

a Calculated for the first 10 s of conversion.

b *k*_c,g_ = *k*_app_/[Au]_g_, where [Au]_g_ represents its concentration in terms of g L^−1^ and *k*_c,m_ = *k*_app_/[Au]_mol_, where [Au]_mol_ represents its concentration in terms of mol L^−1^.

The *k*_app_ of the reaction as a function of ACA-1 concentration was studied by taking a fixed 60 μL volume of the catalyst solution, but varying its initial concentration from 500 to 2000 mg L^−1^ (Fig. S6†). In all the cases, the ln(*C*/*C*_0_) plot profile was observed to be linear, indicating the pseudo first order nature of the reaction. It is evident that the *k*_app_ increases almost linearly with increasing catalyst concentration having an intercept at the *x*-axis at ∼3 nmol of the catalyst. This indicates that a minimum threshold concentration of the catalyst is required for observable catalytic reduction of 4-nitrophenol. The reusability of ACA-1 up to 5 cycles was studied by sequential addition of the reactants into the reaction solution after the completion of each cycle. The time taken for complete reduction of 4-nitrophenol during the cycles 1 to 5 was found to be 15, 100, 120, 150, and 210 s, respectively (Fig. 3d). It can be noted that the TOF_max_ of the second cycle was found to be 16 350 h^−1^ and that of the fifth cycle was observed to be 14 580 h^−1^. The data analysis from the second cycle onwards was performed starting from 3 s, as the addition of reactants was done to an already existing catalyst solution. This requires 1 to 2 s to show the absorbance of 4-nitrophenol during this period correctly. Hence, the TOF_max_ was observed at 3 s for the second to fifth cycles, as opposed to 1 s for the first cycle (Fig. S7†). The decrease in activity over the successive cycles can be attributed to the presence of 4-aminophenol and by-products formed in the previous cycles. It can additionally be noted that the TOF_max_ values from the second cycle onwards are comparable to the NaBH_4_-stabilized Au nanoparticles reported in the literature.^30^

### Catalytic activity and stability of Au nanoclusters towards H_2_ generation from the ammonia borane–sodium borohydride mixture

**Fig. 4:**
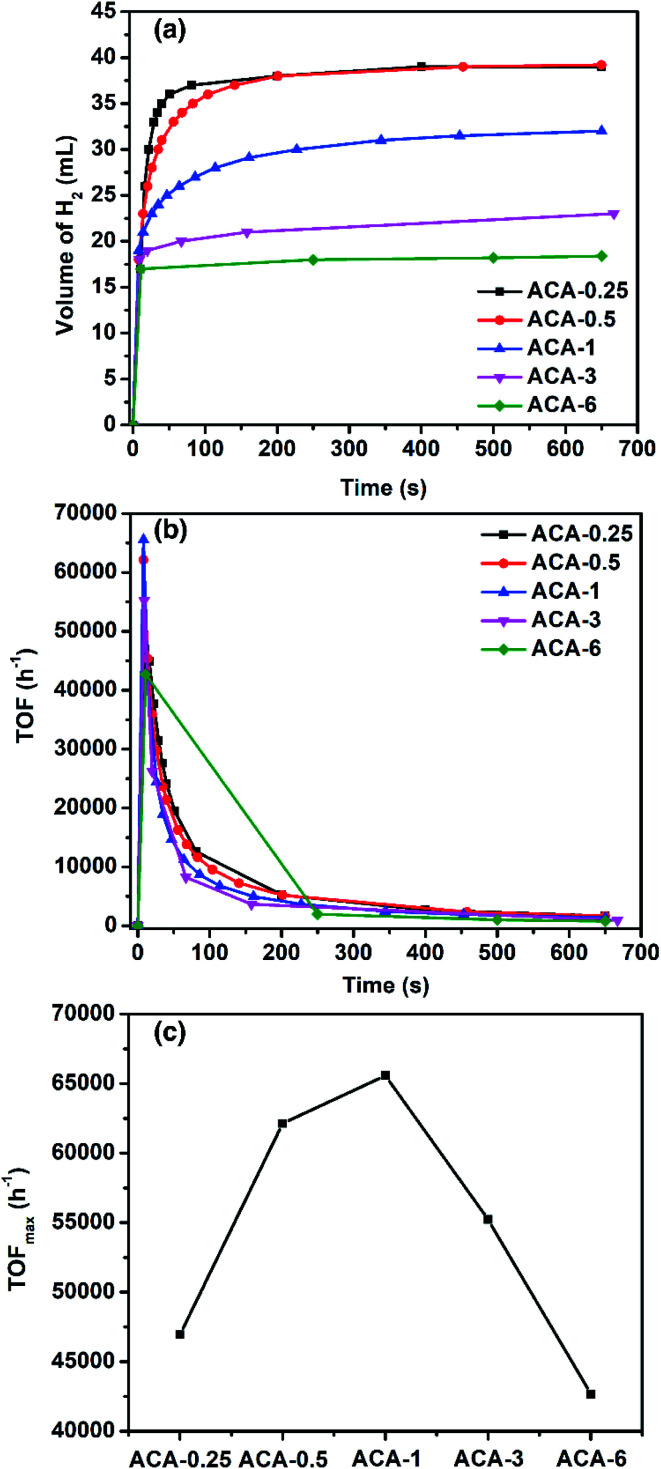
(a) Volume of H_2_ produced as a function of time for various ACA catalysts. Here 2 : 1 molar ratio of ammonia borane to sodium borohydride (10 mg ammonia borane and 6.2 mg sodium borohydride) was dissolved in 2 mL water and added to 50 mg of ACA-1, which was stirred at 735 rpm. (b) TOF as a function of time for various ACA catalysts. (c) The TOF_max_ obtained for various ACA catalysts.

To demonstrate the versatility and ascertain high activity of the synthesized Au nanoclusters, their catalytic efficacy was also studied towards hydrogen generation from ammonia borane. When ammonia borane and NaBH_4_ were individually used, the ACA-0.5 catalyst generated ∼100% hydrogen output but the reaction rate was slow. However, when a mixture of ammonia borane and NaBH_4_ (2 : 1 molar ratio) was used, the rate of H_2_ production was found to be higher (Fig. S8 and S9†). Such a synergistic effect between ammonia borane and NaBH_4_ for H_2_ generation has been known in the literature.^44^Fig. 4a shows the amount of H_2_ generated as a function of time for various ACA catalysts. The total volume of H_2_ generated depends on the gold content in the catalyst: the lower the gold content the more the volume of H_2_ generated. For instance, ACA-0.25 and ACA-0.5 produced 37.5 mL, while ACA-6 produced only 17.5 mL of H_2_ gas. The turn-over frequency corresponding to the catalyst compositions was plotted as a function of time (Fig. 4b). Although, the volume of H_2_ produced in the case of ACA-1 is 32 mL, its TOF_max_ was found to be the highest among the other ACA catalysts (Fig. 4c). A high value of 65 500 h^−1^ was observed for the ACA-1 catalyst under the experimental conditions. Such a high activity for gold has not been reported for H_2_ generation from ammonia borane and the activity has been enhanced to a comparable level to popular candidates such as Pd, Ru, and Rh.^45–49^

In order to gain insights into the mechanism of H_2_ generation from an ammonia borane–NaBH_4_ mixture, the kinetic studies were performed using the ACA-1 catalyst. The order of the reaction with respect to the H_2_ source mixture and catalyst was found to be zero and 1 (the rate was determined from the second point of production), respectively (Fig. S10 and S11†). In methanol, the volume of H_2_ evolved was 17 mL and the rate of H_2_ hydrogen production was less. Thus the solvent plays a crucial role and it can be envisaged in two ways: (1) the ligand, *i.e.*, citric acid molecules that encompass gold clusters are highly soluble in water and get washed away quickly and as a result more catalytic sites are available for H_2_ generation, whereas the same ligands are practically insoluble in methanol and as a result both the volume and rate of H_2_ production is less. (2) The source of hydrogen also comes from the solvent, suggesting that the mechanism of H_2_ production proceeds through solvolysis. Since the reaction follows zero order with respect to the ammonia borane concentration, the adsorption of ammonia borane on the catalyst surface is the rate-limiting step in the hydrolysis reaction. The ammonia borane/sodium borohydride mixture reacts with the surface of the catalysts leading to the surface-hydrogen species, which then combine to form H_2_ gas. The values of rate of H_2_ evolution for ACA catalysts are given in Table 3.

**Table 3:** Catalytic activity of ACA catalysts towards H_2_ generation. The catalyst attributes have been given in Table 2

Sample	TOF_max_ (h^−1^)	Initial rate^a^ (mL s^−1^)	Overall rate^a^ (mL s^−1^)
ACA-0.25	47 000	1.7	0.444
ACA-0.5	62 500	2.25	0.189
ACA-1	65 500	2.375	0.126
ACA-3	55 000	2.0	0.944
ACA-6	42 500	1.545	1.545

a Rate of H_2_ evolution, *r*_H_2__ = *k*_eff_[ammonia borane–NaBH_4_]^0^. The rate, *r*_H_2__, was calculated from Fig. 4a. The initial rate was calculated based on the initial two-second time data. The overall rate was calculated based on the time at which H_2_ evolution stopped. In all the experiments, the amount of gold taken was 1.12 mg (600 mg L^−1^).

### Studies on catalyst aggregation in an aqueous solution

**Fig. 5:**
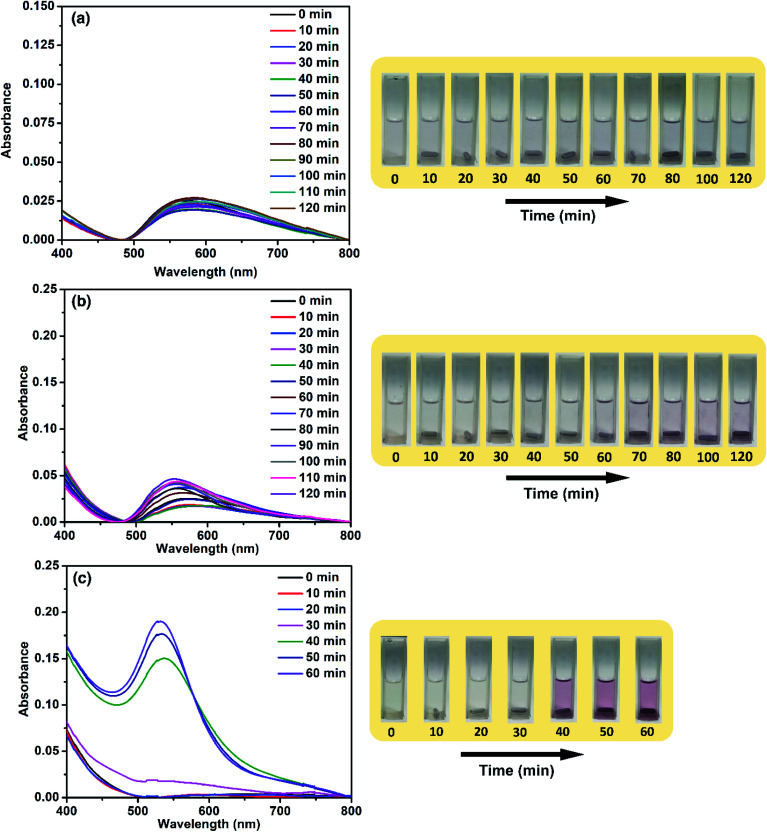
Aggregation dynamics study of Au nanoclusters by monitoring the time-dependent UV-visible spectra of (a) ACA-0.25 (12 000 mg L^−1^), (b) ACA-1 (3000 mg L^−1^), and (c) ACA-6 (500 mg L^−1^) solutions. The panel on the right side shows the digital images of the corresponding solutions for visual observations. For the same aggregation dynamics studies, a 1000 mg L^−1^ of ACA-1 catalyst solution showed no visible color change by 120 min. Hence, the initial concentration of 3000 mg L^−1^ of ACA-1 catalyst solution was used.

From this study so far, it is clear that the catalytic activity of ACA catalysts reaches a peak during the initial stages of the reaction. Also, the activity of the ACA catalyst towards 4-nitrophenol reduction decreases for the scenarios of low concentration of the catalyst and over successive cycles. To gain further insight into this, the aggregation phenomenon was monitored by using the time-dependent UV-visible profile of Au nanocluster solutions without the reactants. For ACA-1 and ACA-6, an evolution of peak at around 600 nm was observed, whose intensity increased with time along with a blue shift (Fig. 5). This blue shift correlates with a clear visible characteristic pink coloration of the Au nanoparticles due to the surface plasmon resonance. The time taken for the visible detection of the plasmonic color was dependent on the citric acid content. In ACA-6, the visible plasmonic color was observed by 30 min, while the color was observed by 40 and 50 min for ACA-3 (Fig. S12†) and ACA-1, respectively. In both ACA-0.25, and ACA-0.5 (Fig. S12†), no blue shift of the 600 nm peak was seen, hence no visible plasmonic color was observed up to 120 min. These observations clearly indicate that the citric acid content plays a crucial role in minimizing the growth of Au nanoclusters into plasmonic Au nanoparticles.

The aggregation studies revealed lower stability of Au nanoclusters in solution, indicating the weak capping ability of citric acid. This was ascertained by carrying out the reduction of 4-nitrophenol in methanol, which is a bad solvent for citric acid and NaBH_4_. Interestingly, no reduction of 4-nitrophenol was observed in this case, indicating that citric acid remained at the surface of Au nanoclusters and the reactants did not access the surface active sites. In the case of the ammonia borane reaction, ∼12 mL H_2_ was generated in methanol as opposed to 39 mL in water. It should be noted that ammonia borane is soluble in methanol. Thus, the high catalytic activity in water is substantiated by the hypothesis that the ligands over the Au nanoclusters swiftly move away from the surface, paving the path for reactants to approach and access catalytically active sites of ‘infant’ Au nanoclusters. We coined the term ‘infant’ Au nanoclusters owing to the following reasons: (i) the active sites are preserved in the as-prepared kinetically stable solid state; (ii) the weakly capped citric acid ligands swiftly move away in the aqueous medium to give rise to infant Au nanoclusters that unleash record high catalytic activity during the initial stage of the reaction before maturing to a more stable state thereupon.^50^ Also, the instant availability of high concentration of the surface active sites can be evidenced by the absence of any induction period in the 4-nitrophenol reaction. Although, in the literature, restructuring of the surface atoms is proposed to be the cause for the induction period, this hypothesis is still under exploration.^51–53^ However, other studies have attributed the decrease in the induction period to the increase in the catalyst concentration.^54^ It is noteworthy that in our study, no induction period was observed when the ACA-1 catalyst solutions have been freshly prepared and introduced into the reaction medium. This strongly indicates the ready availability of the surface active sites in the as-prepared catalyst solution. The catalyst solutions were aged up to 50 min and then studied further for their catalytic efficacy. The rate of 4-nitrophenol reduction was found to decrease when the waiting (aging in solution) period was increased from 10 s to 40 min, for which the complete conversion required 40 s (Fig. S13†). An induction period of 10 s was observed when the waiting period was further increased to 50 min and the complete conversion in this case required 120 s. These studies revealed that the infant Au nanoclusters can grow into a matured state in the aqueous phase (even without any other added reagents), wherein the coalescence of nanoclusters led to the loss in the concentration of surface active sites and subsequent decrease in their catalytic activity. In the case of hydrogen generation studies, the rapid deterioration in the activity of the catalysts with increasing gold content could be due to the aggregation or fouling of the nanoclusters by the polyborazelyne byproduct. In other words, the lower the citric acid content in the catalyst composition, the easier the catalyst fouling/aggregation, while the higher citric acid content retained the catalysts' activity for a relatively longer period of time. Nevertheless, all the catalyst compositions have shown a very high early catalytic activity.

### Estimation of surface active sites

Adsorption of 2-mercaptobenzimidazole (2-MBI) on the surface of noble metal nanosystems can yield a number of catalytically active sites.^55–59^ The 2-MBI adsorption was performed to quantify the surface active sites by using a liquid chromatography (LC) technique (Fig. S14 and S15†). It should be noted that there exists a possibility for the growth of Au nanoclusters and/or their ligand-mediated etching during the experiment. However, to obtain a semi-quantitative perspective, it is assumed that there does not take place any aggregation as well as ligand-mediated etching of the Au nanoclusters and thereby the catalytically active sites remain intact for the reaction with 2-MBI. In a typical experiment, 100 μL of 1000 mg L^−1^ aqueous solution of ACA-0.25, ACA-0.5, and ACA-1 samples was equilibrated with 1 mL of aqueous 2-MBI for 1 h duration. Other samples, namely, ACA-3 and ACA-6 contained a considerable amount of partially unreacted AuCl_3_, which interfered with the experiment, and hence were not considered for this analysis. The adsorption isotherm plotted for ACA-1 suggests that the Langmuir model was being followed with a surface saturation of 12.04 ± 0.63 nmol of 2-MBI (Fig. S16†). It should be noted that the amount of Au in the nanoclusters used in the study was 11.1 nmol, and hence the molecular ratio of 2-MBI adsorbed to the initial gold content is 1.05. Considering only the surface Au atoms by taking the average cluster size to be 1 nm, this ratio gets increased to 1.19 for ACA-1 (see the ESI†). A similar observation of a higher Au–S coordination number was also experimentally determined through extended X-ray absorption fine structure by Fernandez *et al.* for 1.4 nm gold nanoparticles capped with dodecanethiol.^60^ Such a higher coordination was attributed to multiple Au–S bonds at the surface Au atoms. In the case of ACA-0.25 and ACA-0.5, the ratio of 2-MBI adsorbed to the initial gold content was found to be 0.48 and 0.89, respectively. The reasons for the unexpectedly low surface saturation values are unclear at this stage, however, they may be partially attributed to the presence of excess citric acid in spite of 2-MBI being a stronger ligand. Nevertheless, the study has clearly indicated that the surface active sites of ACA-1 are completely available for catalysis, which corroborates a very high TOF for 4-nitrophenol reduction.

Besides, we also showed that two other factors such as grinding time and nature of the reducing agent/ligand can also influence the catalytic reaction rate. Firstly, we studied the 4-nitrophenol reduction using ACA-1 (20 min) that took 35 s to complete, which is 2.5 times slower than that of the 10 min ground sample (Fig. S17†). This result can be correlated with the gentle increase in the particle size and crystallinity due to the excessive grinding of the reaction mixture. Secondly, two more reducing agents such as tartaric acid and ascorbic acid in the place of citric acid were employed, however, the resultant compositions were found to be ineffective, when compared with ACA-1 (Fig. S18 and S19†).

## Conclusions

To conclude, a facile and elegant room temperature synthesis of weakly capped infant Au nanoclusters is reported that sets a new gold standard for the catalytic activity of Au-based systems by employing the benchmark reaction of 4-nitrophenol reduction and catalytic H_2_ generation from the ammonia borane–NaBH_4_ mixture. Our study alleviates the requirement of any separate ligand removal step and provides fundamental insights into the synthetic approach that yields “infant” Au nanoclusters. These infant Au nanoclusters can be stabilized in the solid citric acid matrix over a period of three months, when stored at 4 °C under an inert atmosphere. When introduced in the reaction medium, Au nanoclusters demonstrated extraordinarily high catalytic activity in the early stages, before gradually maturing into a less active state. The cyclability studies for 4-nitrophenol showed that the catalysts possessed significant activity over 5 cycles, a behavior similar to that of the supported catalysts. On the other hand, the Au nanoclusters exhibited very high early catalytic activity towards hydrogen generation from the ammonia borane–NaBH_4_ mixture, before undergoing aggregation/catalyst fouling. Given the record high catalytic activity of the infant Au nanoclusters in the solution phase reactions, we anticipate new possibilities of these catalysts in the gas phase catalytic reactions as well.^6,26^ Furthermore, we believe that the unconventional synthetic methodology reported here would lead to a plethora of avenues both from the synthetic and application points of view.

## Conflicts of interest

There are no conflicts to declare.

## Supplementary Material

NA-002-D0NA00639D-s001

NA-002-D0NA00639D-s002
