# Supplementary material for: New gold standard: weakly capped infant Au nanoclusters with record high catalytic activity for 4-nitrophenol reduction and hydrogen generation from an ammonia borane–sodium borohydride mixture
Source: Nanoscale Adv. 2020 Sep 28;2(11):5384–95. doi: 10.1039/d0na00639d (PMC9417437; doi:10.1039/d0na00639d)
Supplement: NA-002-D0NA00639D-s001 [file NA-002-D0NA00639D-s001.pdf]

Electronic Supplementary Material (ESI) for Nanoscale Advances.
This journal is © The Royal Society of Chemistry 2020

Supplementary Information

New Gold Standard: Weakly-Capped Infant Au Nanoclusters with Record High Catalytic Activity for 4-Nitrophenol Reduction and Hydrogen Generation from Ammonia Borane-Sodium Borohydride Mixture

Dinabandhu Patra,[†] Srinivasa Rao Nalluri,[†] Hui Ru Tan,[‡] Mohammad S. M. Saifullah,[*,‡] Ramakrishnan Ganesan,[*,†] Balaji Gopalan[*,†]

[†] Department of Chemistry, Birla Institute of Technology and Science (BITS) Pilani, Hyderabad Campus, Jawahar Nagar, Kapra Mandal, Hyderabad-500078, India

[‡] Institute of Materials Research and Engineering, A*STAR (Agency for Science, Technology, and Research), 2 Fusionopolis Way, #08-03 Innovis, Singapore 138634, Republic of Singapore

[*] Corresponding authors' email addresses: ram.ganesan@hyderabad.bits-pilani.ac.in; gbalaji@hyderabad.bits-pilani.ac.in; saifullahm@imre.a-star.edu.sg

Table S1. The concentrations and volumes of the ACA-1 catalyst solution and reactants used in the catalytic reduction of 4-nitrophenol and cyclability studies.

Cycle	Catalyst volume added (μL) (1000 mg/L ACA-1)	Concentration of 4-nitrophenol (mM)	Volume of 4-nitrophenol added (mL)	Concentration of sodium borohydride (mM)	Volume of sodium borohydride added (mL)	Total reaction volume (mL)
1	60	0.14	1	14	1	2.06
2	0	0.14	0.05[a]	14	0.05[b]	2.16
3	0	0.14	0.0525 [a]	14	0.0525 [b]	2.265
4	0	0.14	0.055 [a]	14	0.055 [b]	2.375
5	0	0.14	0.0575 [a]	14	0.0575 [b]	2.49

[a] The given volumes have been taken from a 6 mM 4-nitrophenol stock solution.

[b] The given volumes have been taken from a 600 mM sodium borohydride stock solution.

Table S2. Average size and standard deviation in size for various synthesized nanoclusters.

Sample	Avg. Size (nm)	Std. Dev. (nm)	Polydispersity (%)
ACA-0.25	0.8	0.10	6.5
ACA-1	1.0	0.10	12.8
ACA-6	1.9	0.60	30.8
ACA-1 20 min	1.3	0.10	10.3

Table S3. Comparison of $AuCl_3$ percentage reduction in ACA-1, as a function of grinding time, determined through XPS and titration method.

Grinding time (min)	Percentage reduction of Au^{3+}	
	From XPS	From titration
2	82.5	83.3
4	84.1	87.5
6	87.2	87.5
8	91.5	91.6
10	94.9	95.8

Table S4. Comparison of $AuCl_3$ percentage reduction in ACA-1, ACA-3, and ACA-6 determined through XPS and titration method.

Sample	Percentage reduction of Au^{3+}	
	From XPS	From titration
ACA-1	94.9	95.8
ACA-3	87.6	91.6
ACA-6	81.7	79.2

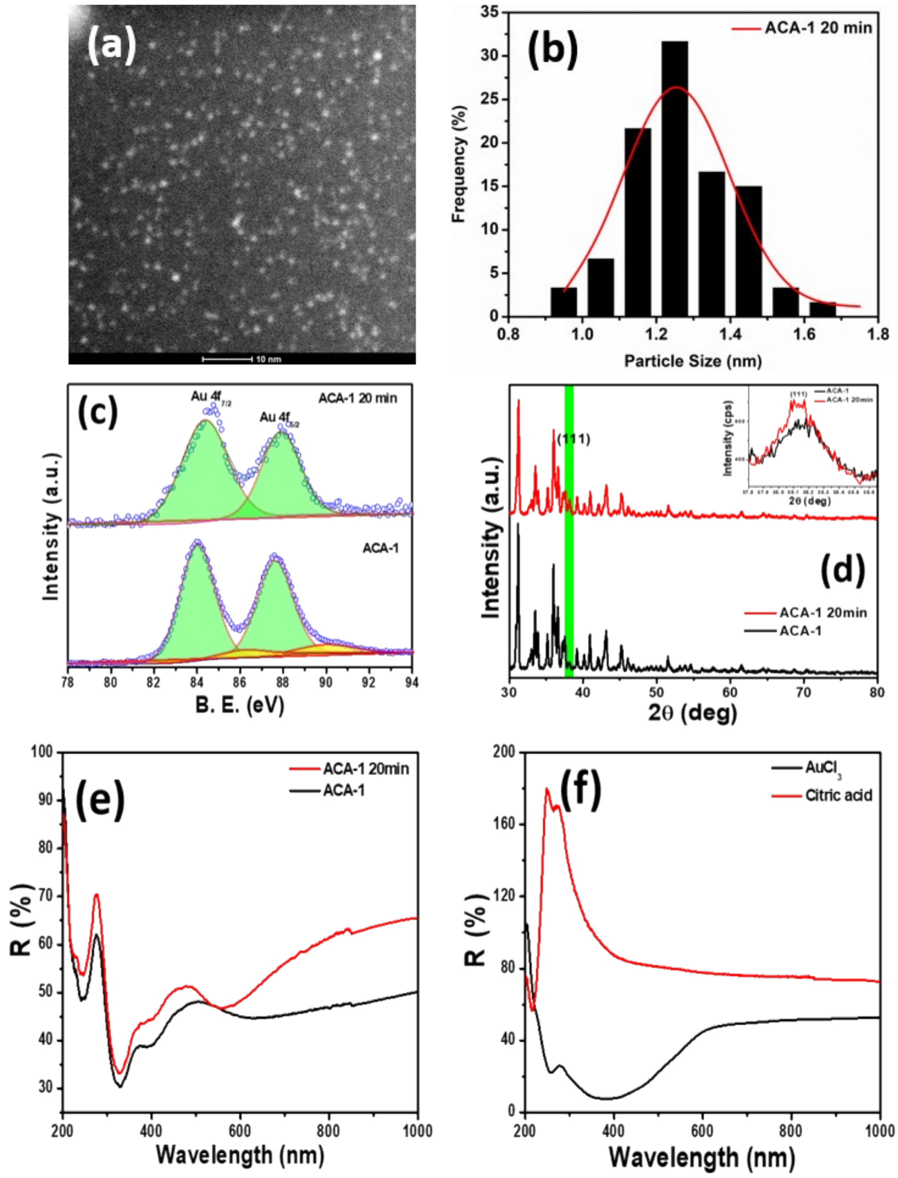

Fig. S1. Data summary for ACA-1 (20 min): (a) A representative STEM-HAADF image, (b) size distribution as determined from (a), (c) narrow scan XPS, (d) XRD, and (e) DRS plots of ACA-1 composition synthesized over 20 min of grinding. In the plots (c), (d), and (e), the data of ACA-1 are also given for easy comparison. (f) DRS spectra of control samples citric acid and KI mixed $AuCl_3$.

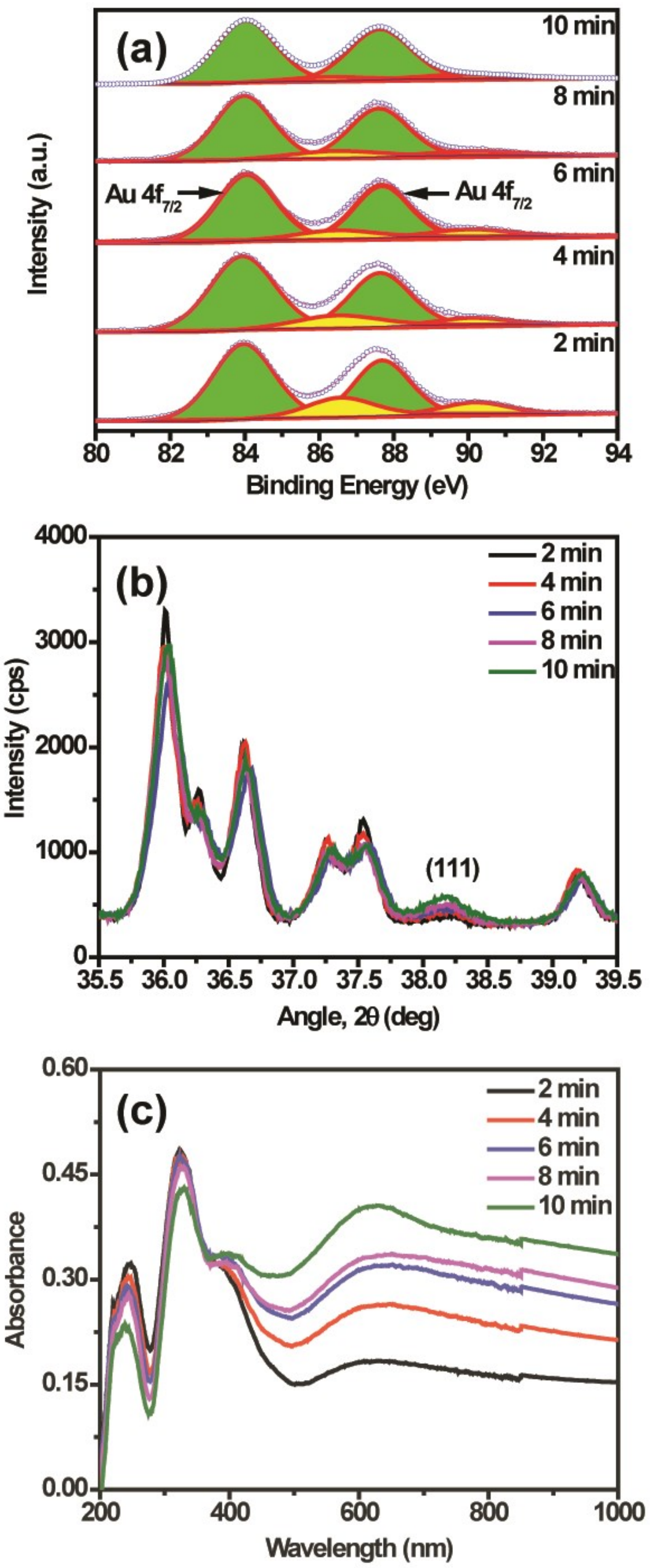

Fig. S2. Time dependent (a) narrow scan XPS, (b) XRD, and (c) solid state DRS analyses for ACA-1 composition prepared by grinding of $AuCl_3$ and citric acid at different time intervals.

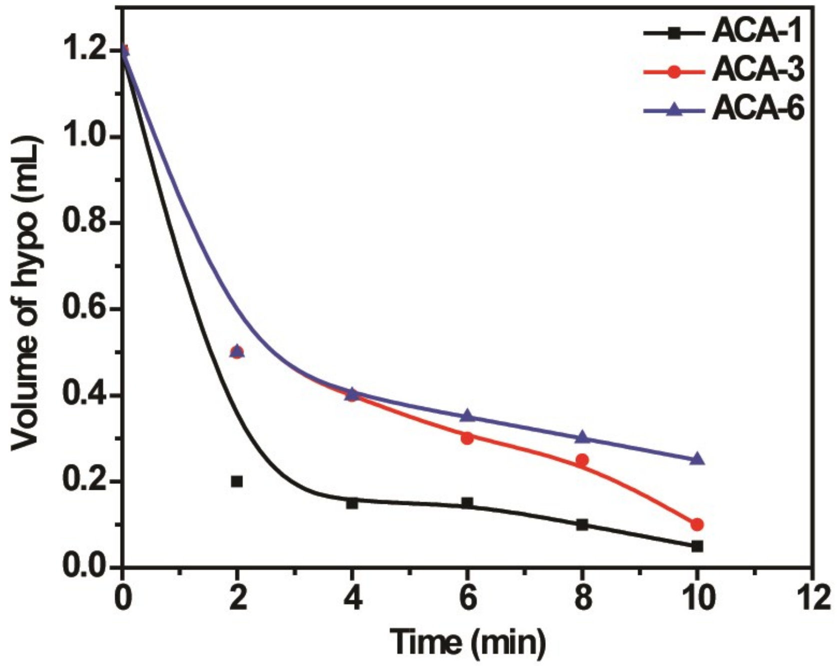

Fig. S3. Time-dependent redox titration of ACA-1, ACA-3 and ACA-6 compositions (synthesized at different time intervals) against 0.625 mM sodium thiosulfate solution. Stock solutions for ACA-1, ACA-3, and ACA-6 were made by dissolving 26, 9.4, and 5.2 mg of the solid sample in 50 mL of water, respectively, such that the total gold content in each case matches closely with the control titration (1 mg $AuCl_3$ solution (12 wt% of water) added to 50 mL of water). For each titration, 2 mL of the sample solution taken from the stock solution was used.

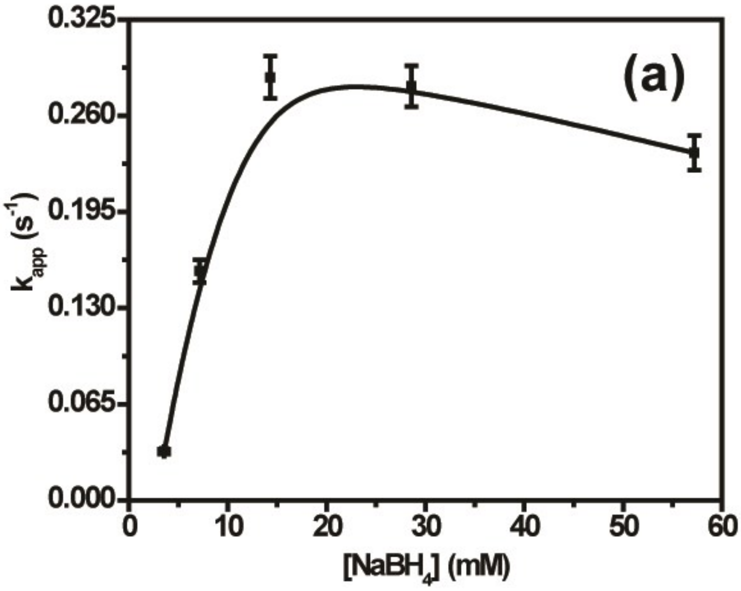

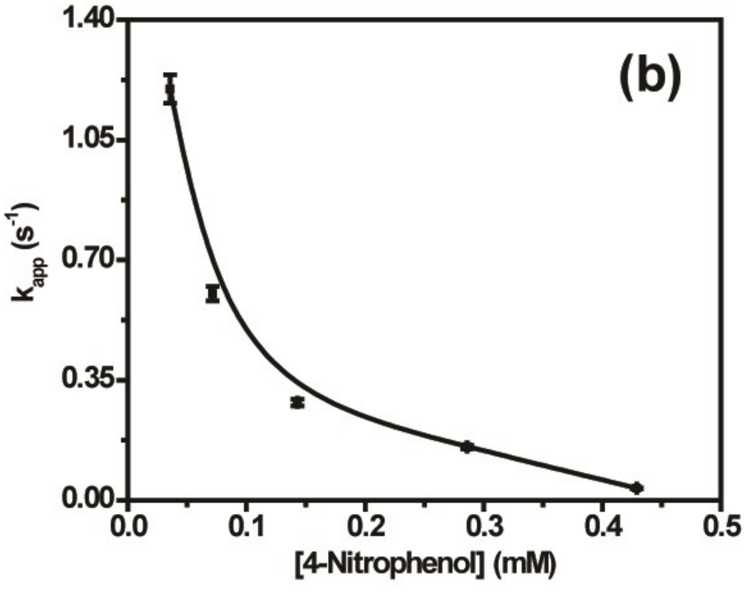

Fig. S4. The dependence of k_{app} on the initial concentration of (a) sodium borohydride by taking 0.14 mM of 4-nitrophenol and (b) 4-nitrophenol by taking 1:100 ratio of 4-nitrophenol to sodium borohydride. In all the reactions, 60 μL of 1000 mg/L concentration catalyst solution was used. The lines are given as a guide to the eyes. The trend indicates the Langmuir-Hinshelwood mechanism.

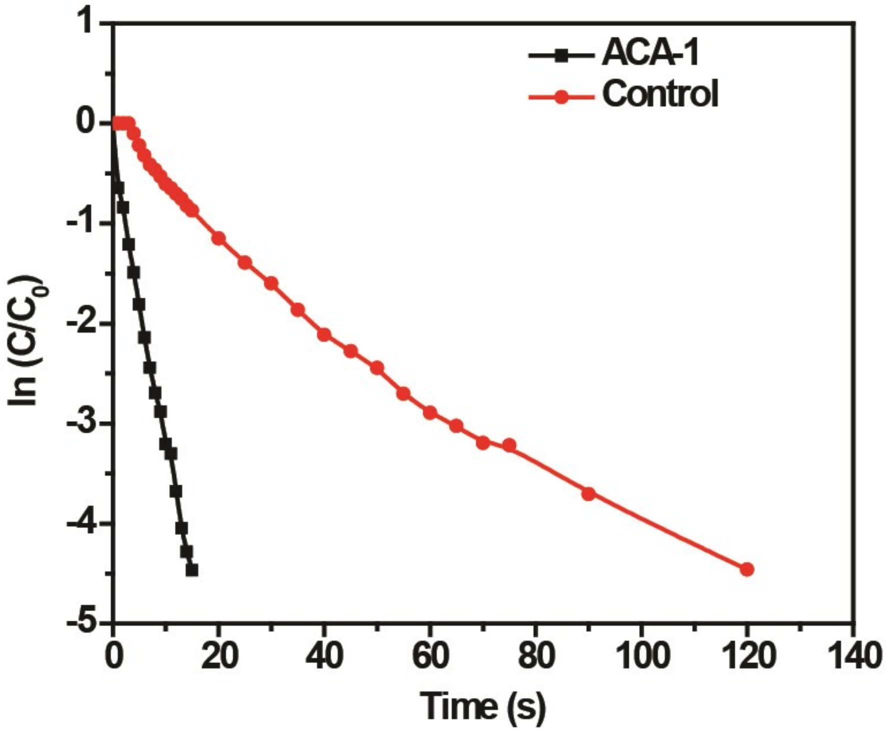

Fig. S5. Comparison of the catalytic activity of ACA-1 with the control experiment.

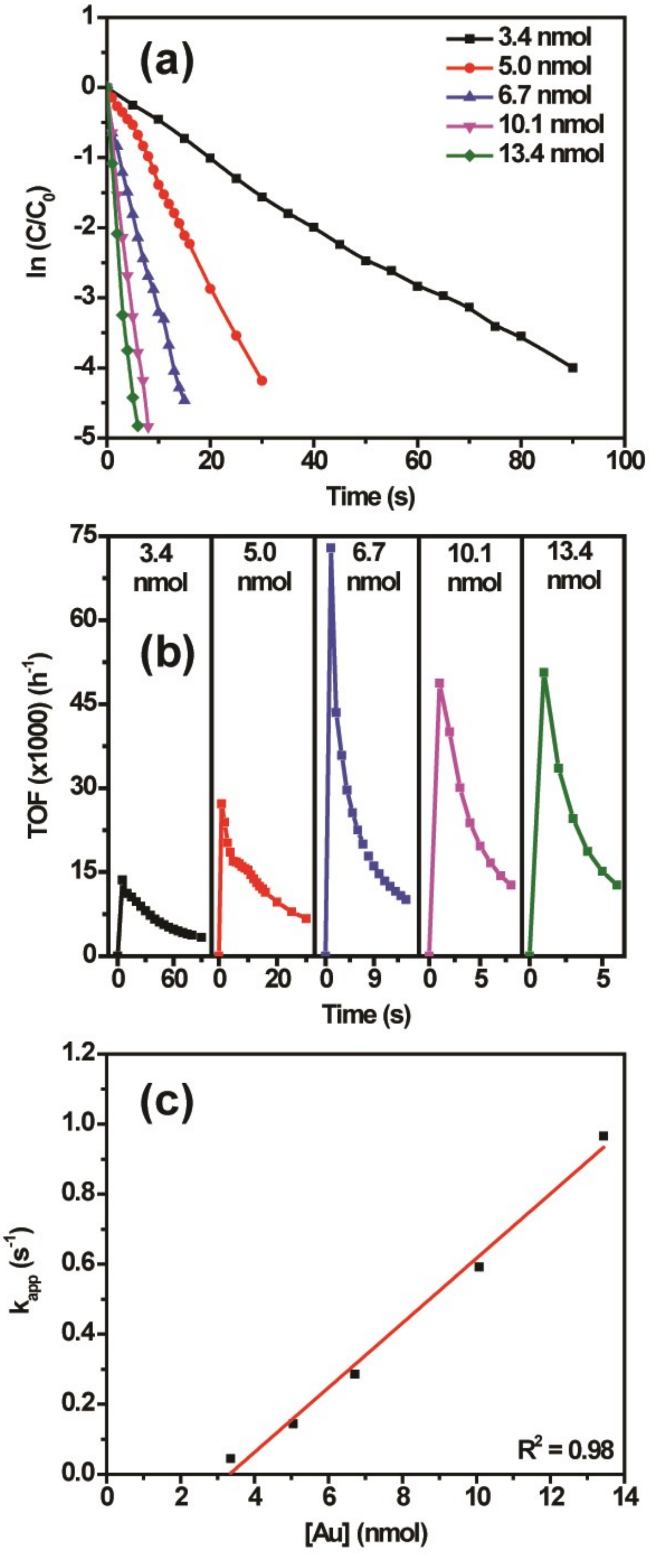

Fig. S6. Effect of ACA-1 concentration on catalytic activity towards 4-nitrophenol reduction: (a) $\ln(C/C_0)$ *versus* time plot, (b) TOF *versus* time, and (c) dependence of k_{app} as a function of Au concentration. It can be noted that a fixed 60 µL volume of catalyst solution taken from stock having initial concentrations as 500, 750 1000, 1500, 2000 mg/L has been used in 2 mL reaction volume containing 0.14 and 14 mM of 4-nitrophenol and sodium borohydride, respectively. The actual amount of Au used in reactions have been indicated in nmol.

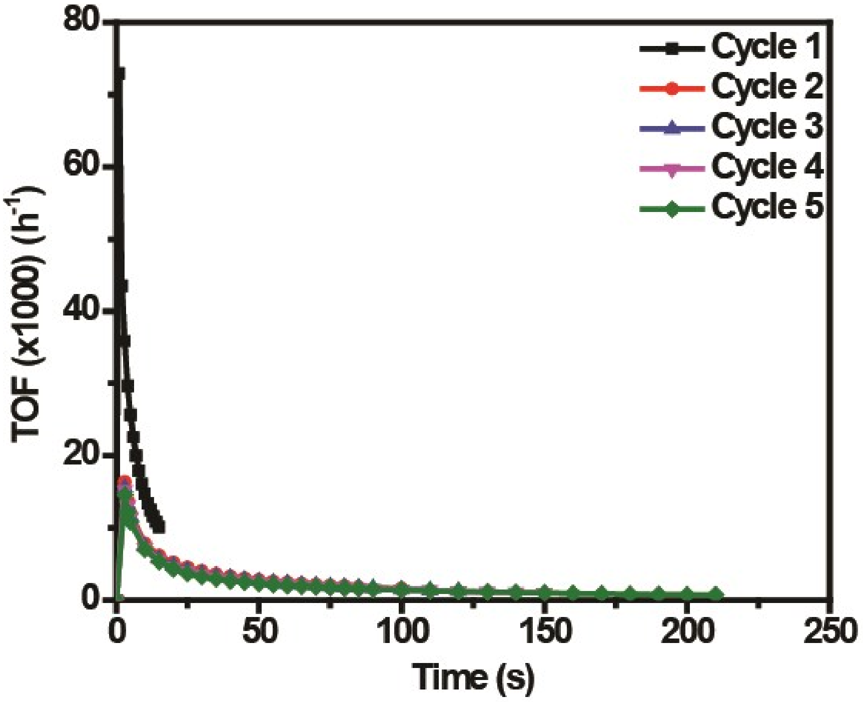

Fig. S7. TOF *versus* time for cyclability studies using ACA-1 catalyst.

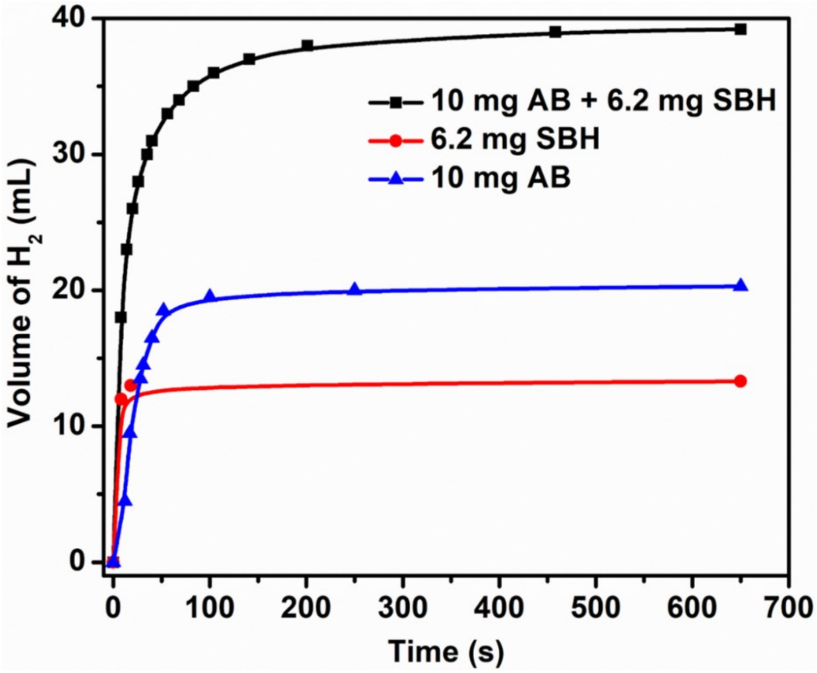

Fig. S8: Volume of H_2 produced as a function of time with pure ammonia borane, $NaBH_4$ and 2:1 molar ratio mixture of ammonia borane and $NaBH_4$, respectively. The abbreviations AB and SBH in the legends represent ammonia borane and sodium borohydride, respectively.

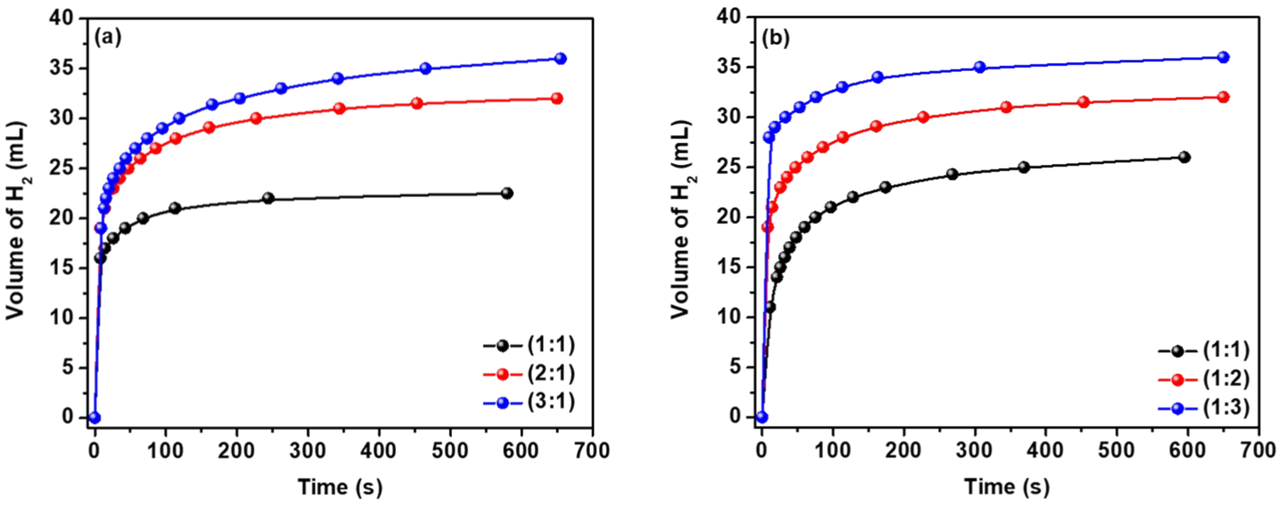

Fig. S9. (a) Synergistic effect of sodium borohydride on ammonia borane for H_2 generation. The reaction conditions were: ammonia borane amount was kept constant as 6.2 mg and sodium borohydride amount was varied from 5 mg to 15 mg in 2 mL of water. This solution was added to 50 mg of solid ACA-1 and was stirred at 735 rpm. (b) Synergistic effect of ammonia borane on sodium borohydride for H_2 generation. The reaction conditions were: sodium borohydride amount was kept constant as 10 mg and ammonia borane amount was varied from 3 mg to 9 mg, the solution was then added to 50 mg of ACA-1 catalyst and the contents were stirred at 735 rpm.

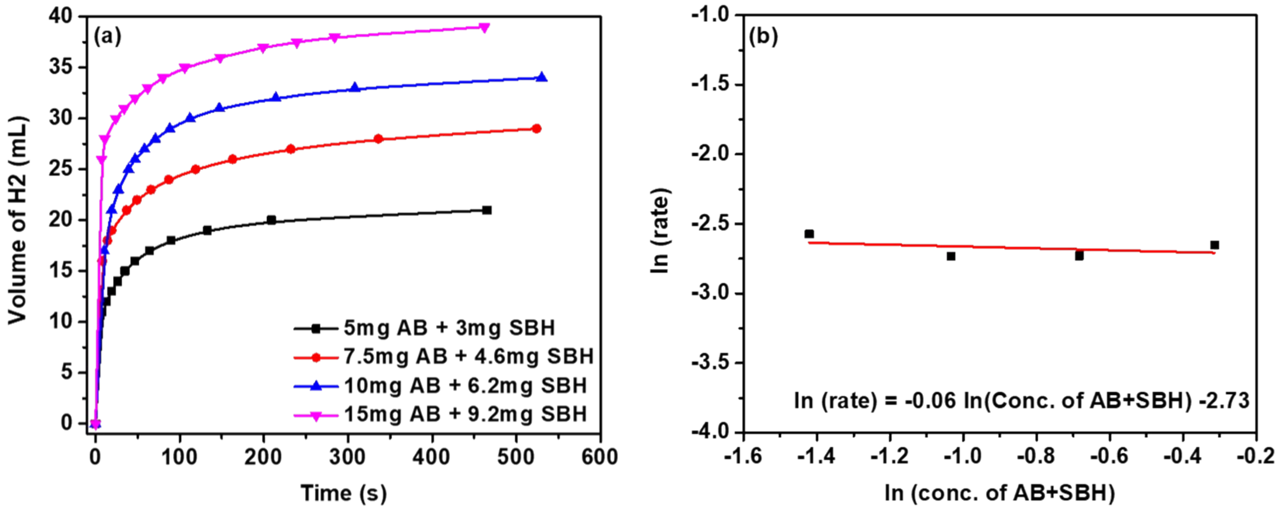

Fig. S10. (a) Volume of H_2 evolved as a function of time for various concentration of hydrogen source. (b) Plot of ln(rate) vs ln(hydrogen source) reaction. The reaction conditions were indicated amounts of mixed hydrogen source was dissolved in 2 mL water and added into the 50 mg solid ACA-1 and the contents were stirred at 735 rpm. The rate was calculated from second point. The abbreviations AB and SBH in the legends represent ammonia borane and sodium borohydride, respectively.

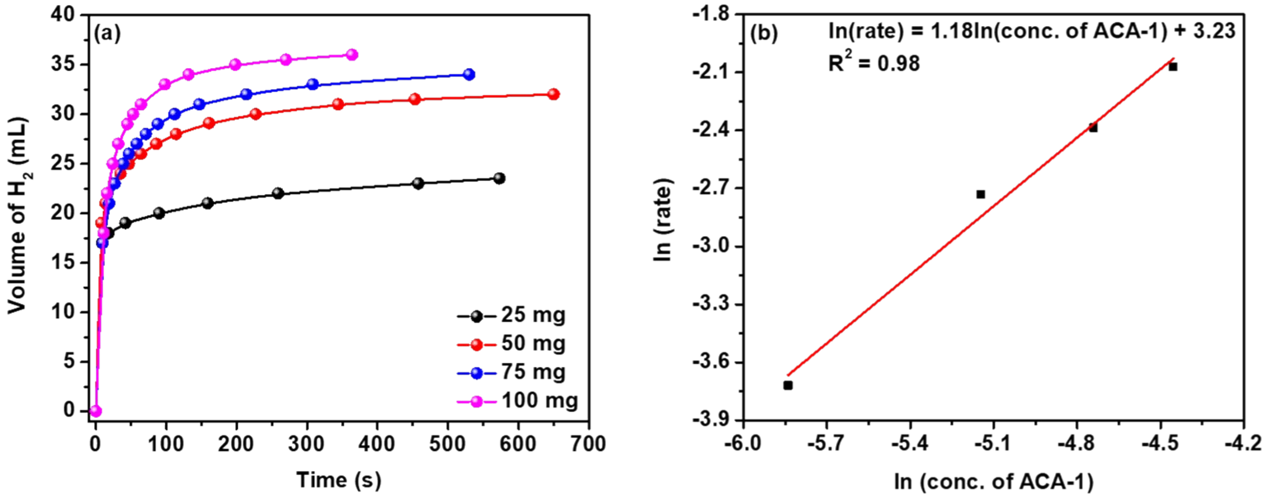

Fig. S11. (a) Plot of H_2 evolved as a function of time for various catalyst concentration. The reaction conditions were: a constant amount of 2:1 ratio of ammonia borane to sodium borohydride (10 mg ammonia borane & 6.2 mg sodium borohydride) was dissolved in 2 mL water and added into various amount of ACA-1 catalyst. The contents were stirred at 735 rpm. (b) Plot of ln(rate) against ln([ACA-1]). The rate was calculated from second point.

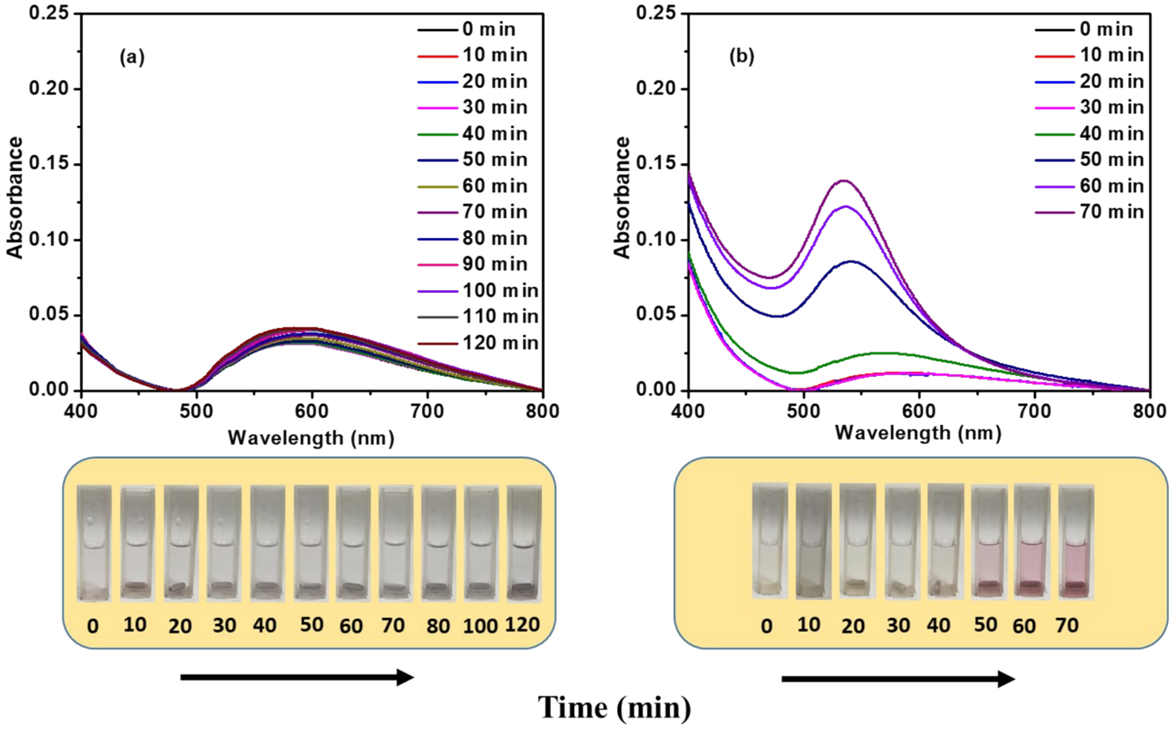

Fig. S12. Aggregation dynamics study of Au nanoclusters by monitoring the time dependent UV-visible spectra of (a) ACA-0.5 (6000 mg/L) and (b) ACA-3 (1000 mg/L) solutions. The bottom panels show the digital images of the corresponding solutions for visual observations.

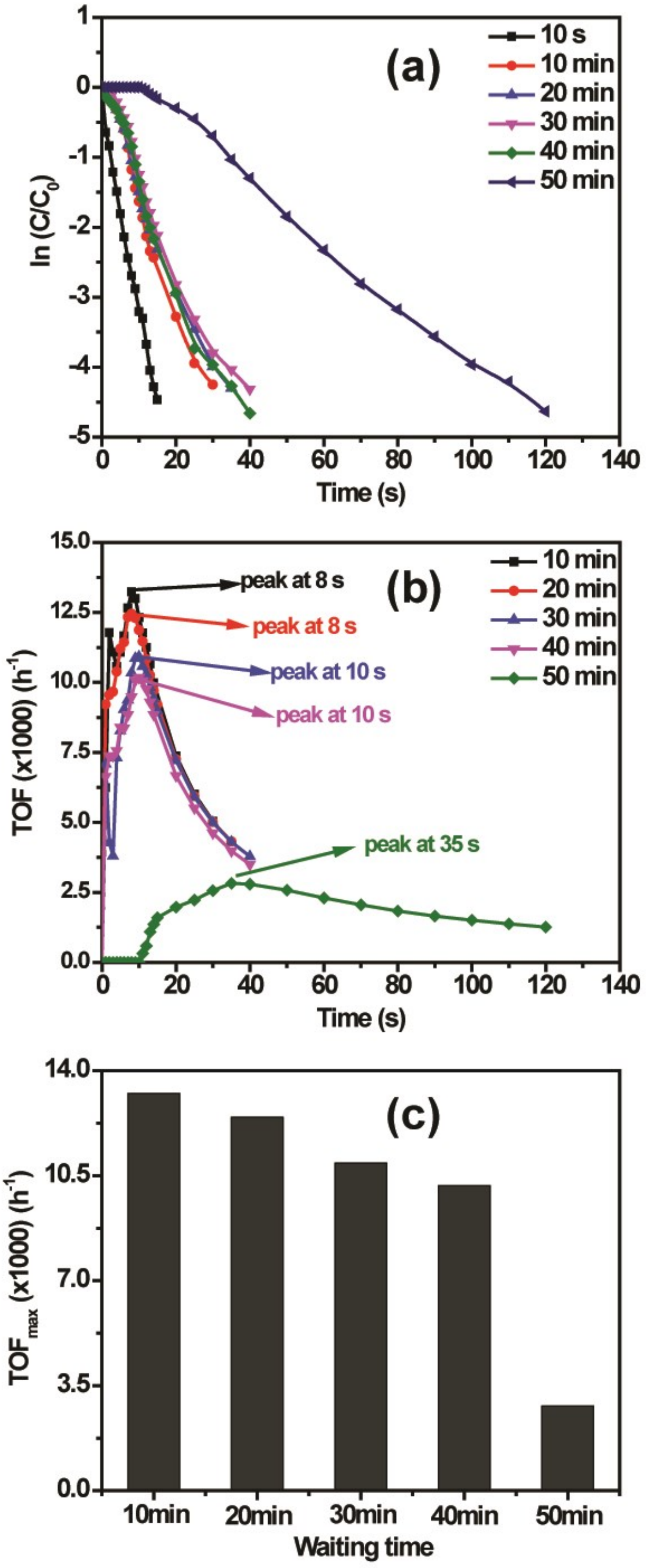

Fig. S13. Effect of waiting period of the catalyst solution from the time of its preparation to introduction into the reaction medium. (a) $\ln(C/C_0)$ *versus* time plot for 4-nitrophenol reduction with ACA-1 catalyst introduced at different waiting times, and the corresponding (b) TOF *versus* time plot, and (c) the bar diagram of TOF_{max}.

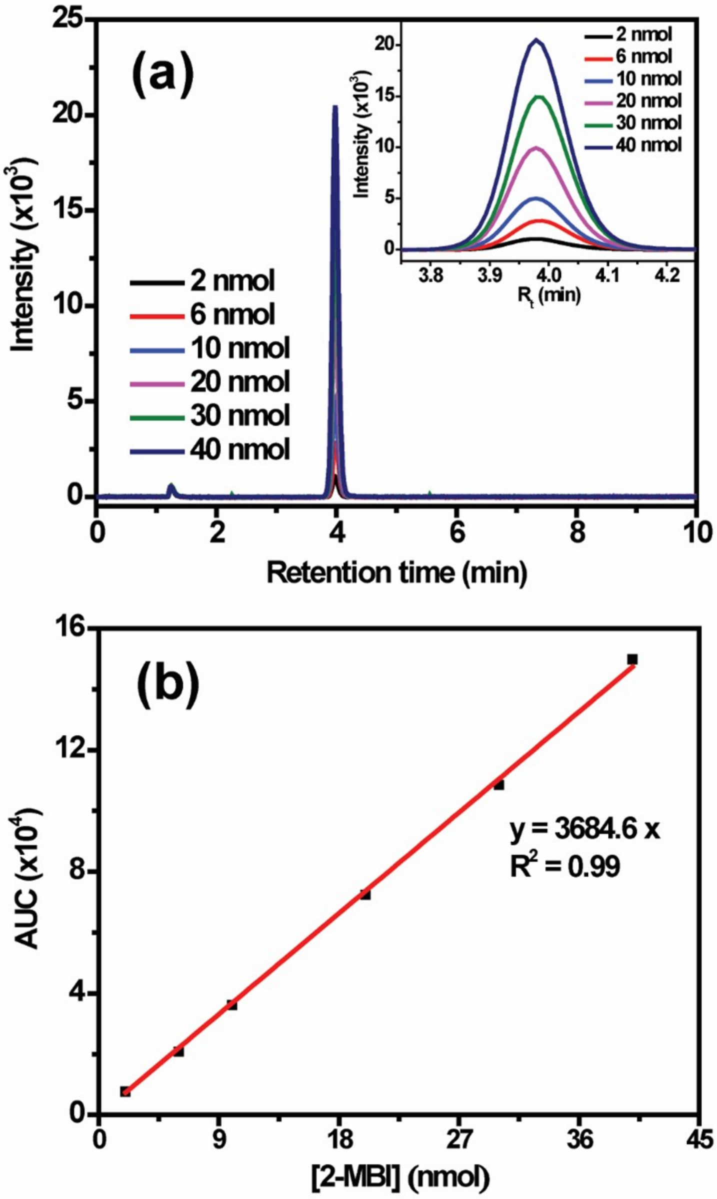

Fig. S14. (a) Liquid chromatography (LC) profiles pertaining to the calibration of 2-MBI. The inset shows the zoomed in peak profile of 2-MBI. (b) Linear fitting of area under the curve (AUC) *versus* 2-MBI concentration. In each LC run, 100 µL of 1000 mg/L citric acid solution was treated for 1 h with 2 mL of 1:1 water-methanol mixture containing the indicated initial nmol of 2-MBI.

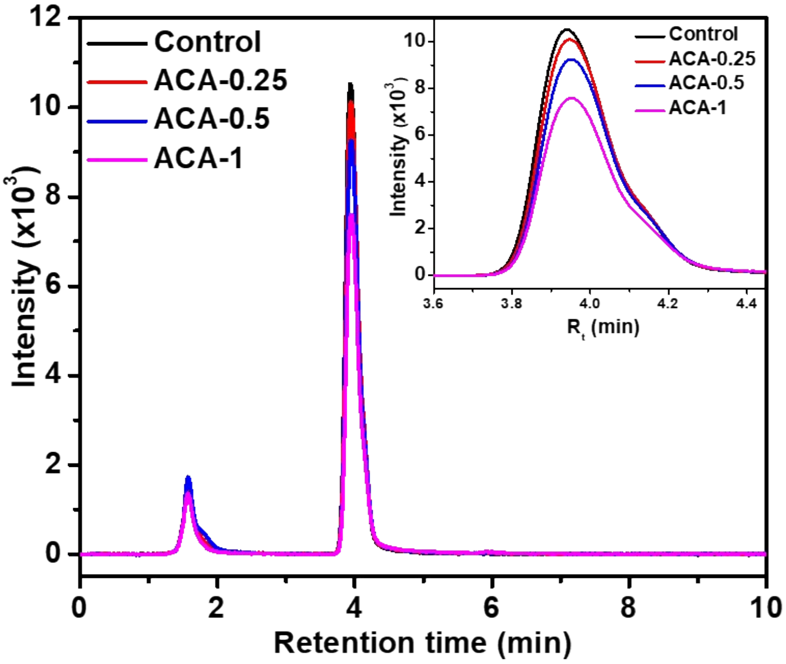

Fig. S15. Estimation of surface active sites from the LC profile measurements of unadsorbed 2-MBI after its treatment with the ACA catalysts. The zoomed in peak profile of 2-MBI for the various scans are shown in the inset.

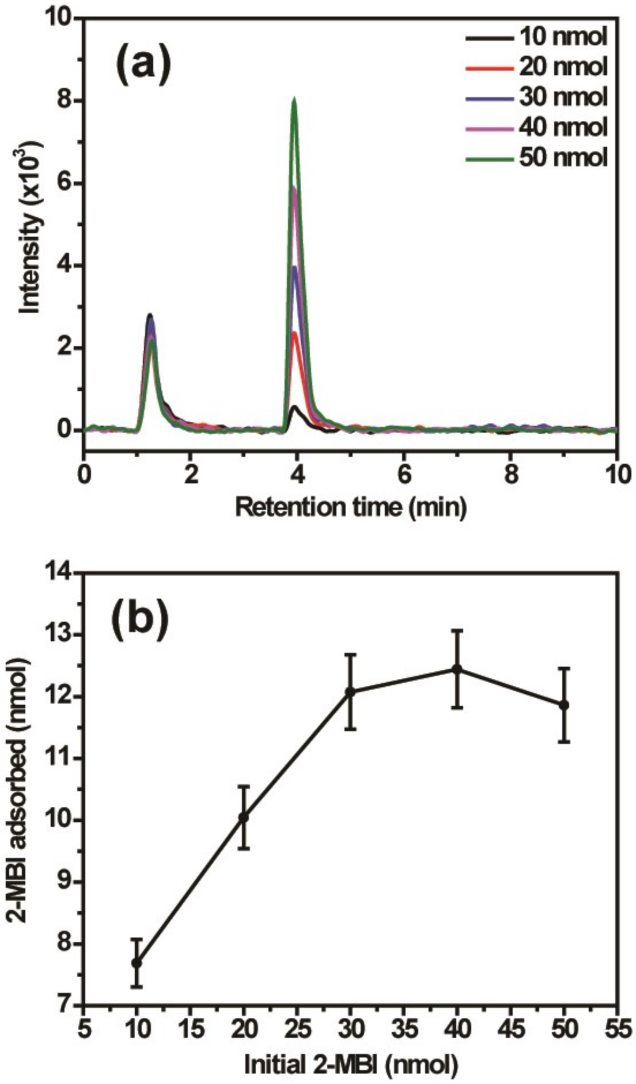

Fig. S16. (a) LC profiles for various concentrations of 2-MBI treated with a fixed concentration of ACA-1 solution. In each of the case, 100 μL of 1000 mg/L ACA catalyst aqueous solution was treated for 1 h with 2 mL of 1:1 water-methanol mixture containing the indicated initial nmol of 2-MBI. (b) Plot showing the amount of 2-MBI adsorbed *versus* initial concentration of 2-MBI.

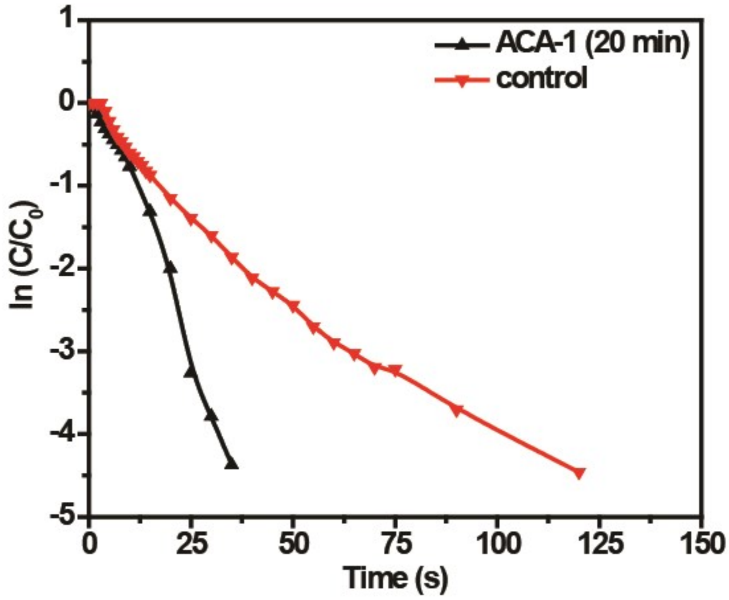

Fig. S17. The ln(C/C_0) plot *versus* time corresponding to the catalytic 4-nitrophenol reduction using ACA-1 (20 min). The control experimental data is given along with for comparison.

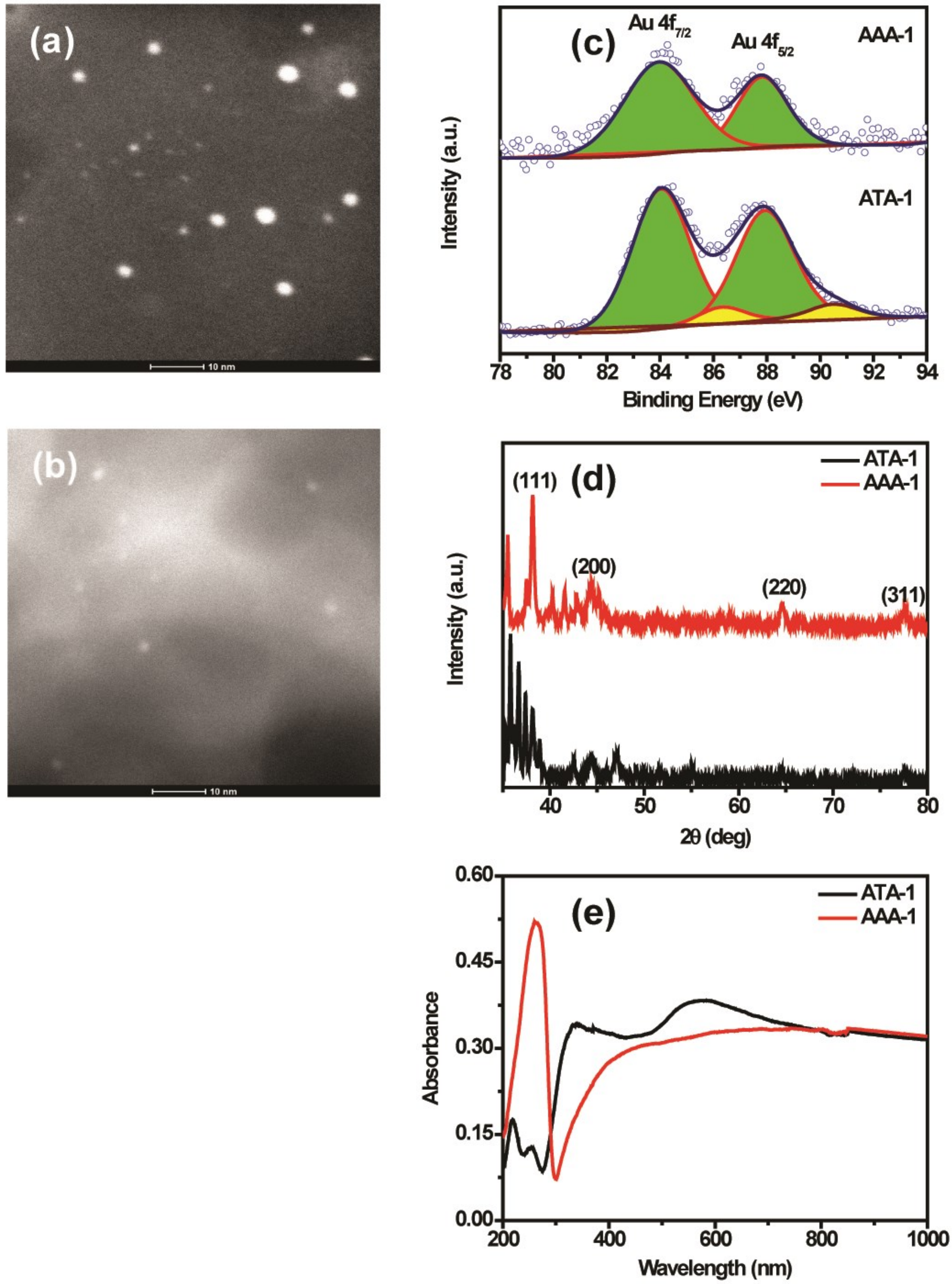

Fig. S18. Data summary for AAA-1 and ATA-1: Representative STEM-HAADF images of (a) ATA-1 and (b) AAA-1; (c) Au *4f* narrow scan XPS, (d) XRD, and (e) DRS plots.

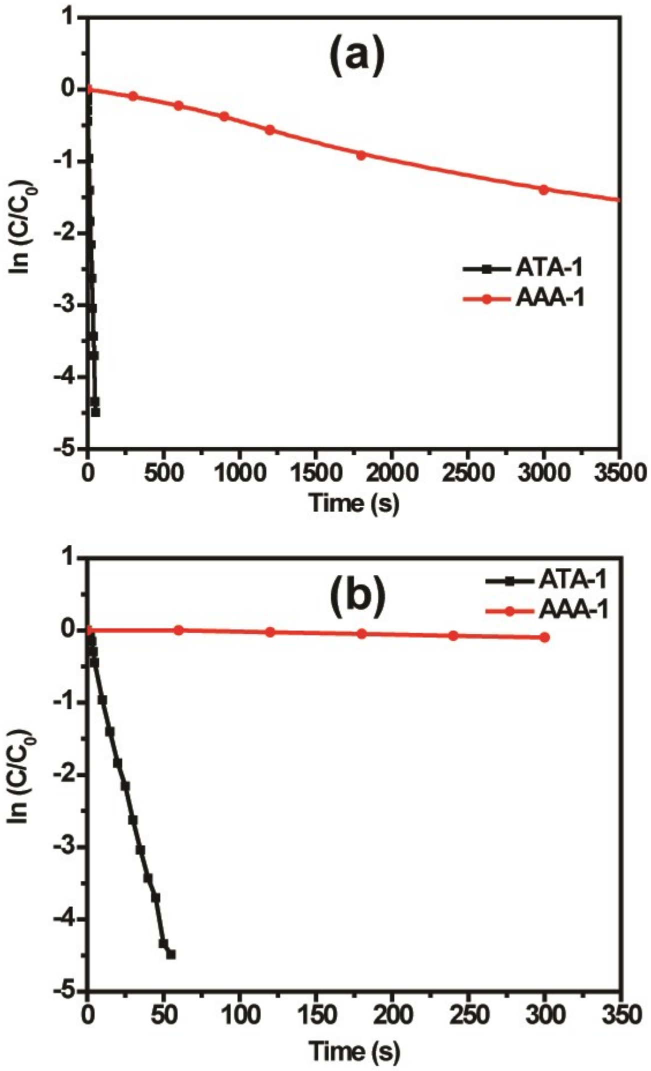

Fig. S19. (a) The ln(C/C_0) plot *vs*. time corresponding to the catalytic 4-nitrophenol reduction using AAA-1 and ATA-1 showing up to 3500 s. (b) The same plot has been zoomed in up to 350 s for clarity with respect to ATA-1.

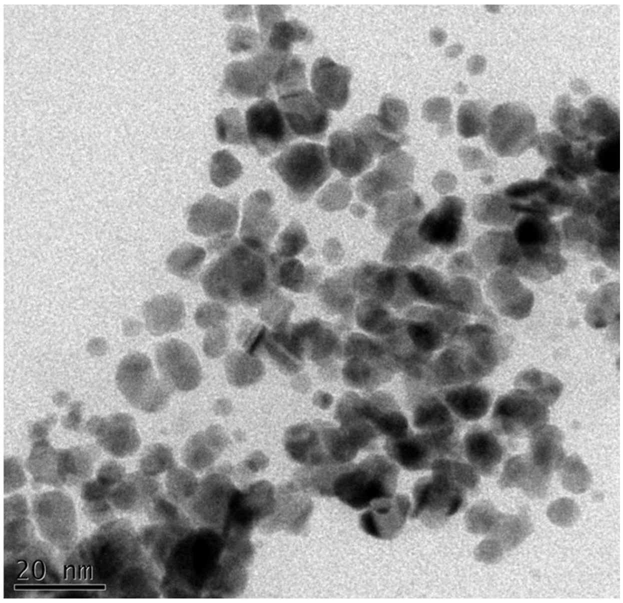

Fig. S20. A representative transmission electron microscopy image ACA-1 sample prepared over the Cu grid through the conventional solution casting and drying method. Comparing with STEM images, it is clear that growth of Au nanoclusters takes place during conventional sampling.

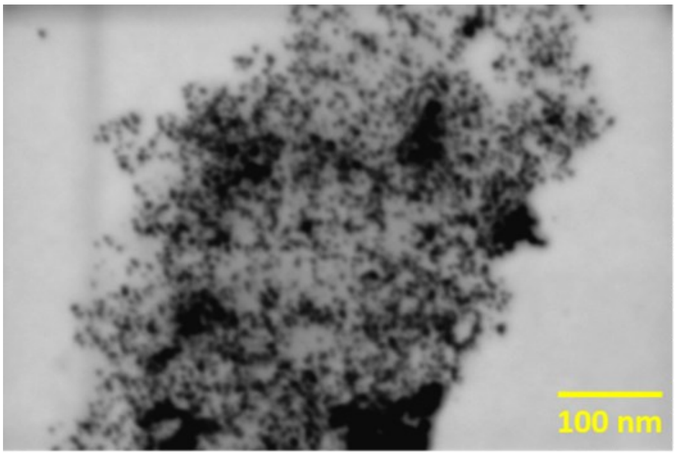

Fig. S21. A representative bright field STEM image of recovered ACA-1 catalyst after 4-nitrophenol reduction reaction.

Calculations:

I. TOF calculation:

(a) TOF_{max} calculation with ACA-1 catalyst for 4-nitrophenol reduction.

Molecular weight of $AuCl_3$ is 303.325 g/mol.

Molecular weight of Au is 196.97 g/mol.

The as-synthesized ACA-1 catalyst contains 5.3 mg of $AuCl_3$ in 150 mg citric acid that corresponds to 3.44 mg of Au.

For catalysis, 60 µL of 1000 mg/L ACA-1 aqueous solution was added to 2 mL of a solution containing 0.14 mM 4-nitrophenol and 14.3 mM sodium borohydride.

The number of moles of Au used for catalysis can then be calculated as follows:

1000 µL of catalyst stock solution contains 1 mg of ACA-1. This corresponds to 0.02205 mg of Au ($\frac{3.44}{156}$) in 1 mL of water.

Therefore, 60 µL solution contains $\frac{60 * 0.02205}{1000}$ = 0.001323 mg of Au.

Number of moles of Au present in 60 µL of ACA-1 catalyst solution = $\frac{0.001323}{196.97}$ = 6.716 nmol.

The reaction medium contains 0.286 µmol of 4-nitrophenol.

Hence, TOF = $\frac{Initial\ mol\ of\ 4-nitrophenol\ -\ mol\ of\ 4-nitrophenol\ present\ at\ time\ (t)}{Mol\ of\ Au\ used\ in\ catalysis\ \times\ time\ (t)}$

For example, TOF_{max} calculated after 1 s for ACA-1 = $\frac{(0.286\ -\ 0.15)\ \mu mol}{6.716\ nmol\ \times\ 1}$ = 20.25 s^{-1} = 72,900 h^{-1}

(b) TOF_{max} calculation with ACA-1 catalyst for hydrogen generation:

The amount of catalyst used for each reaction is 50 mg.

The amount of Au present in 50 mg catalyst = 1.147 mg.

Number of moles of Au present in 1.147 mg = $\frac{1.147}{196.97}$ = 0.005823 mmol.

19 mL H_2 was generated in 8 s.

Therefore, TOF = $\frac{Moles\ of\ hydrogen\ gas\ generated}{Mol\ of\ Au\ used\ in\ catalysis\ \times\ time\ (t)}$

TOF_{max} = $\frac{19/22400}{(0.005823/1000)\ \times\ 8}$ = 18.21 s^{-1} = 65,549 h^{-1} (assuming ideal gas)

II: Calculation of surface active sites for ACA samples using 2-MBI:

It is assumed that the number of 2-MBI molecules adsorbed corresponds to the number of available surface active sites in Au nanoclusters.

Let us consider the average diameter of Au nanoclusters to be 1 nm (1×10^{-7} cm)

Then, the volume of each nanocluster = $\frac{4}{3}\pi r^3 = 5.233 \times 10^{-22}$ cm^3

Mass of a single nanocluster = volume × density = $5.233 \times 10^{-22} \times 19.32$ g/cm^3 (assuming bulk density of Au) = 1.011×10^{-20} g

No. of moles of Au present in a single nanocluster = mass of the nanocluster / molecular weight of Au = 1.011×10^{-20} / 196.97 = 5.133×10^{-23} moles

Total number of Au atoms in a single nanocluster = Total no. of moles of Au in a single nanocluster × Avogadro number. = $5.133 \times 10^{-23} \times 6.02 \times 10^{23} = 30.9$

Total number of surface Au atom calculation

The number of surface Au atoms was calculated by subtracting the number of Au atoms below the surface layer of the sphere from the total number of Au atoms in a single nanocluster. To estimate the number of atoms *below* the surface layer of the sphere, we start by defining the subsurface radius equal to the nanoparticle radius minus one *diameter* of the element. The radius of a single Au atom is 0.136 nm. Therefore, a nanocluster with radius of 0.5 nm (1 nm diameter) would have subsurface radius, $r_{sub\text{-}surface}$ = 0.5 nm – (2 × 0.136) nm = 0.228 nm. Repeating the calculation as shown above, but using the subsurface radius, the total number of Au atoms present below the surface is calculated to be 2.9.

Hence, the number of surface Au atoms = 30.9 – 2.9 = 28

This shows that the dispersion of surface Au atoms in 1 nm diameter cluster to be, 28/30.9 × 100 = 90.61%.

Now, for the ACA-1 catalyst, the amount of Au was 0.002205 mg (2.205×10^{-6} g) for the 2-MBI adsorption study.

The total mass of Au atoms at the surface of 2.205×10^{-6} g of Au = 0.9061×2.205 x 10^{-6} g

= 1.9959×10^{-6} g.

This corresponds to 1.9959×10^{-6} g / 196.97 g.mol^{-1} = 10.13 nmol of Au atoms in the surface.

Experimentally, the total amount of 2-MBI adsorbed over ACA-1 is determined to be 12.04 ± 0.63 nmol.

Thus, the ratio of adsorbed 2-MBI molecules to surface Au atoms = 12.04/10.13 = 1.19.
